# Effects of *Mycobacterium vaccae* NCTC 11659 and Lipopolysaccharide Challenge on Polarization of Murine BV-2 Microglial Cells

**DOI:** 10.3390/ijms25010474

**Published:** 2023-12-29

**Authors:** Luke W. Desmond, Evan M. Holbrook, Caelan T. O. Wright, Cristian A. Zambrano, Christopher E. Stamper, Adam D. Bohr, Matthew G. Frank, Brendan K. Podell, Julie A. Moreno, Andrew S. MacDonald, Stefan O. Reber, Rogelio Hernández-Pando, Christopher A. Lowry

**Affiliations:** 1 Department of Integrative Physiology, University of Colorado Boulder, Boulder, CO 80309, USA; luke.desmond@colorado.edu (L.W.D.); evan.holbrook@colorado.edu (E.M.H.); caelan.wright@colorado.edu (C.T.O.W.); cristian.zambrano@colorado.edu (C.A.Z.); christopher.stamper@colorado.edu (C.E.S.); adam.bohr@colorado.edu (A.D.B.); matt.frank@colorado.edu (M.G.F.); 2 Center for Neuroscience, University of Colorado Boulder, Boulder, CO 80309, USA; 3 Department of Microbiology, Immunology, and Pathology, Colorado State University, Fort Collins, CO 80523, USA; brendan.podell@colostate.edu; 4 Prion Research Center, College of Veterinary Medicine and Biomedical Sciences, Colorado State University, Fort Collins, CO 80523, USA; julie.moreno@colostate.edu; 5 Department of Environmental and Radiological Health Sciences, College of Veterinary Medicine and Biomedical Sciences, Colorado State University, Fort Collins, CO 80523, USA; 6 Center for Healthy Aging, Colorado State University, Fort Collins, CO 80523, USA; 7 Lydia Becker Institute of Immunology and Inflammation, University of Manchester, Manchester M13 9NT, UK; andrew.macdonald@manchester.ac.uk; 8 Laboratory for Molecular Psychosomatics, Department of Psychosomatic Medicine and Psychotherapy, Ulm University Medical Center, 89081 Ulm, Germany; stefan.reber@uni-ulm.de; 9 Sección de Patología Experimental, Departamento de Patología, Instituto Nacional De Ciencias Médicas Y Nutrición Salvador Zubirán, Ciudad de México 14080, Mexico; rhdezpando@hotmail.com; 10 Department of Psychology and Neuroscience, University of Colorado Boulder, Boulder, CO 80309, USA; 11 Center for Microbial Exploration, University of Colorado Boulder, Boulder, CO 80309, USA; 12 Department of Physical Medicine and Rehabilitation and Center for Neuroscience, University of Colorado Anschutz Medical Campus, Aurora, CO 80045, USA

**Keywords:** arginase 1, endotoxin, hygiene hypothesis, lipopolysaccharide, LPS, M1, M2, microglia, neuroinflammation, Old Friends

## Abstract

Previous studies have shown that the in vivo administration of soil-derived bacteria with anti-inflammatory and immunoregulatory properties, such as *Mycobacterium vaccae* NCTC 11659, can prevent a stress-induced shift toward an inflammatory M1 microglial immunophenotype and microglial priming in the central nervous system (CNS). It remains unclear whether *M. vaccae* NCTC 11659 can act directly on microglia to mediate these effects. This study was designed to determine the effects of *M. vaccae* NCTC 11659 on the polarization of naïve BV-2 cells, a murine microglial cell line, and BV-2 cells subsequently challenged with lipopolysaccharide (LPS). Briefly, murine BV-2 cells were exposed to 100 µg/mL whole-cell, heat-killed *M. vaccae* NCTC 11659 or sterile borate-buffered saline (BBS) vehicle, followed, 24 h later, by exposure to 0.250 µg/mL LPS (*Escherichia coli* 0111: B4; *n* = 3) in cell culture media vehicle (CMV) or a CMV control condition. Twenty-four hours after the LPS or CMV challenge, cells were harvested to isolate total RNA. An analysis using the NanoString platform revealed that, by itself, *M. vaccae* NCTC 11659 had an “adjuvant-like” effect, while exposure to LPS increased the expression of mRNAs encoding proinflammatory cytokines, chemokine ligands, the *C3* component of complement, and components of inflammasome signaling such as *Nlrp3*. Among LPS-challenged cells, *M. vaccae* NCTC 11659 had limited effects on differential gene expression using a threshold of 1.5-fold change. A subset of genes was assessed using real-time reverse transcription polymerase chain reaction (real-time RT-PCR), including *Arg1*, *Ccl2*, *Il1b*, *Il6*, *Nlrp3*, and *Tnf*. Based on the analysis using real-time RT-PCR, *M. vaccae* NCTC 11659 by itself again induced “adjuvant-like” effects, increasing the expression of *Il1b*, *Il6*, and *Tnf* while decreasing the expression of *Arg1*. LPS by itself increased the expression of *Ccl2*, *Il1b*, *Il6*, *Nlrp3*, and *Tnf* while decreasing the expression of *Arg1*. Among LPS-challenged cells, *M. vaccae* NCTC 11659 enhanced LPS-induced increases in the expression of *Nlrp3* and *Tnf*, consistent with microglial priming. In contrast, among LPS-challenged cells, although *M. vaccae* NCTC 11659 did not fully prevent the effects of LPS relative to vehicle-treated control conditions, it increased *Arg1* mRNA expression, suggesting that *M. vaccae* NCTC 11659 induces an atypical microglial phenotype. Thus, *M. vaccae* NCTC 11659 acutely (within 48 h) induced immune-activating and microglial-priming effects when applied directly to murine BV-2 microglial cells, in contrast to its long-term anti-inflammatory and immunoregulatory effects observed on the CNS when whole-cell, heat-killed preparations of *M. vaccae* NCTC 11659 were given peripherally in vivo.

## 1. Introduction

Stress-related psychiatric disorders, including anxiety disorders, mood disorders, and trauma- and stressor-related disorders, such as posttraumatic stress disorder (PTSD), are a significant burden worldwide [1,2]. Psychosocial stress and chronic low-grade inflammation are considered risk factors for the development of stress-related psychiatric disorders [3,4]. The “Old Friends” hypothesis posits that increasing rates of inflammation, inflammatory diseases, and stress-related psychiatric disorders in industrialized nations are due, at least in part, to reduced exposure to immunoregulatory microorganisms (i.e., microorganisms that promote a balanced expression of regulatory and effector T cells) [5]. Consequently, one strategy to reduce the risk of these conditions would be to use immunoregulatory approaches to mitigate stress-induced inflammatory responses [6,7].

One of the “Old Friends” is *M. vaccae* NCTC 11659, a soil-derived bacterium with anti-inflammatory and immunoregulatory properties that, when given via the subcutaneous or oral route, can prevent inflammatory conditions, such as allergic airway inflammation in a murine model of allergic asthma [8,9,10], or prevent outcomes of stressor exposures that are driven by inappropriate inflammation [6]. Both the effects of *M. vaccae* NCTC 11659 on allergic airway inflammation [10] and its effects on negative outcomes of stressor exposure (e.g., stress-induced colitis and stress-induced exaggeration of anxiety-like or fear-like behaviors [11]) are dependent on regulatory T cells (Treg) in the periphery.

In addition to these effects of *M. vaccae* NCTC 11659 on peripheral immune signaling when given via the subcutaneous or oral route, it also influences innate immune cells within the central nervous system (CNS), i.e., microglia. Immunization with *M. vaccae* NCTC 11659 via the subcutaneous route can, (1) by itself, decrease hippocampal *Nfkbia* and *Nlrp3* mRNA expression and (2) prevent stress-induced microglial priming, as assessed by LPS-induced increases in *Il1b* and *Nfkbia* mRNA expression in freshly isolated hippocampal microglia [12,13,14,15]. However, the direct effects of *M. vaccae* NCTC 11659 on microglia, as might occur following the translocation of the live bacillus across the blood–cerebrospinal fluid barrier, for example, after the intravenous administration of a heat-killed suspension of *Mycobacterium*, as recently conducted using *Mycobacterium indicus pranii* (*Mycobacterium w*) in critically ill COVID-19 patients [16,17] or as recently conducted using the intravenous administration of the Bacillus Calmette–Guérin (BCG) vaccine in macaques [18], have not been studied.

Therefore, we set out to determine the effects of *M. vaccae* NCTC 11659, relative to vehicle control conditions, applied directly to murine BV-2 microglial cells when followed, 24 h later, by an immune challenge with LPS or vehicle control conditions. For a detailed description of the history of the development of *M. vaccae* NCTC 11659 used here, see [19]. For a description of the alternative designations and different preparations and production processes of *M. vaccae* NCTC 11659 used in clinical trials or preclinical studies, see [20].

## 2. Results

### 2.1. Experiment 1: LPS Dose–Response

**Figure 1:**
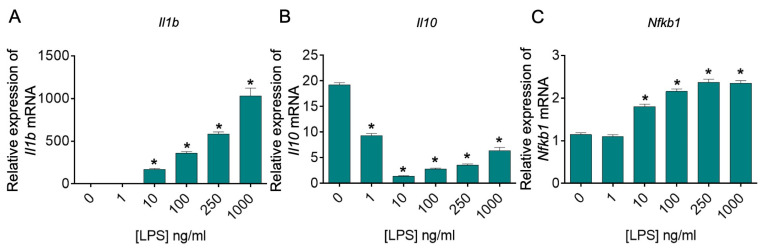
Effects of lipopolysaccharide (LPS) on *Il1b*, *Il10*, and *Nfkb1* mRNA expression in murine BV-2 cells. Murine BV-2 microglial cells were treated with 0, 1, 10, 100, 250, or 1000 ng/mL LPS for 24 h. Cells were harvested, and cDNA was prepared from total RNA. Gene expression was measured using real-time reverse transcription polymerase chain reaction (real-time RT-PCR) and is represented relative to the highest Ct value for each gene using the 2^−∆∆Ct^ method, with *Actb*, which encodes β-actin, as a reference. Data represent mean quantification values (Ct) + SEM, i.e., relative expression of (**A**) *Il1b*, (**B**) *Il10*, and (**C**) *Nfkb1* mRNA. Data were analyzed using a one-way ANOVA followed by Fisher’s LSD test using a single pooled error value, if appropriate, at a two-tailed alpha level of 0.05. * *p* < 0.05, Fisher’s LSD test compared to cell media vehicle (CMV) control group. For all experimental conditions, there were 8 technical replicates (*n* = 8) from *n* = 1 experiment.

Lipopolysaccharide (LPS) is an endotoxin derived from the outer membrane of Gram-negative bacteria, and it is known to induce a robust immune response. In BV-2 cells, LPS increases *Il1b* mRNA expression [21] and induces the release of functional IL-1β, a proinflammatory cytokine [22]. In contrast, LPS decreases *Il10* mRNA and interleukin 10 (IL-10) protein expression, an anti-inflammatory cytokine, in murine BV-2 cells [23]. Finally, LPS enhances NF-κB signaling in BV-2 cells [23], and studies using siRNA knockdown of *Nfkb1*, which encodes NFκB1 p50, suggest that *Nfkb1* plays a key role in the proinflammatory response to LPS and M1 polarization [24]. Therefore, we conducted dose–response studies to evaluate the effects of LPS on *Il1b*, *Il10*, and *Nfkb1* mRNA expression in murine BV-2 cells. LPS induced dose-dependent increases in *Il1b* and *Nfkb1* (Figure 1A,C). In contrast, as expected, LPS induced a decrease in *Il10* mRNA expression (Figure 1B). Taken together, these data demonstrate that, under the conditions used here, LPS dose-dependently induced the expected proinflammatory response and shift toward the M1 immunophenotype of BV-2 cells. We selected the 250 ng/mL LPS concentration for further experiments since it induced a greater-than-2-fold change in all genes studied and induced the maximum response in *Nfkb1* mRNA expression.

### 2.2. Experiment 2: Principal Coordinate Analysis of All Samples Used for the NanoString Platform

**Figure 2:**
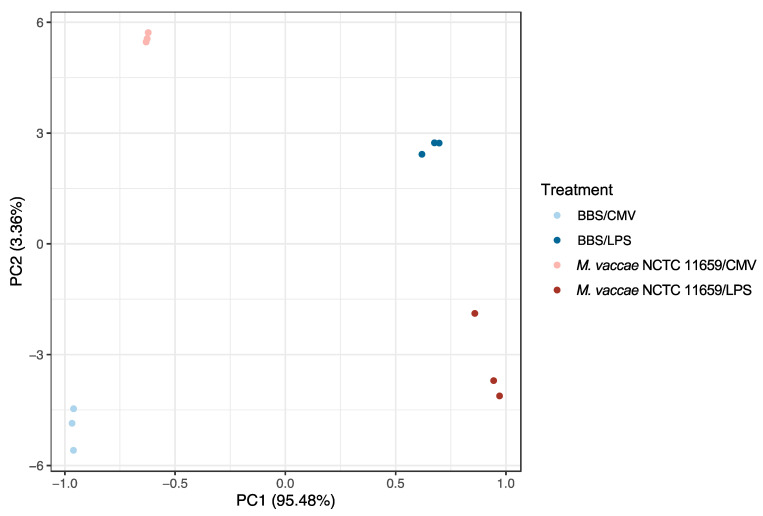
The principal coordinate analysis (PCoA) plot provides a visual representation of the pattern of proximity among all samples in the study based on an analysis of 248 endogenous inflammation-related genes using the nCounter Mouse Inflammation v2 Panel in the NanoString nCounter platform. The figure was generated using the vegan, ecodist, and ggplot2 packages in RStudio. The sample sizes of all experimental conditions were *n* = 3 technical replicates. PC1 explains 95.48% of the variability, and PC2 explains 3.36%. Abbreviations: BBS, borate-buffered saline; CMV, cell media vehicle; LPS, lipopolysaccharide; *M. vaccae* NCTC 11659, *Mycobacterium vaccae* NCTC 11659; PC1, principal coordinate 1; PC2, principal coordinate 2.

The analysis of all samples used for the NanoString platform using a principal coordinate analysis (PCoA) revealed the close clustering of samples within each treatment group (Figure 1). The PC1 axis accounted for 95.48% of the variation, predominantly representing the variation among groups due to treatment with LPS. The PC2 axis accounted for 3.36% of the variation, predominantly representing the variation among groups due to treatment with *M. vaccae* NCTC 11659. Together, PC1 and PC2 accounted for 98.84% of the total variation. An analysis using PERMANOVA revealed significant differences among the groups (*F*_(4,7)_ = 2.69, *p* < 0.001; Figure 2).

### 2.3. Effects of M. vaccae NCTC 11659 on mRNA Expression in BV-2 Microglial Cells Based on Analysis Using the NanoString Platform

**Figure 3:**
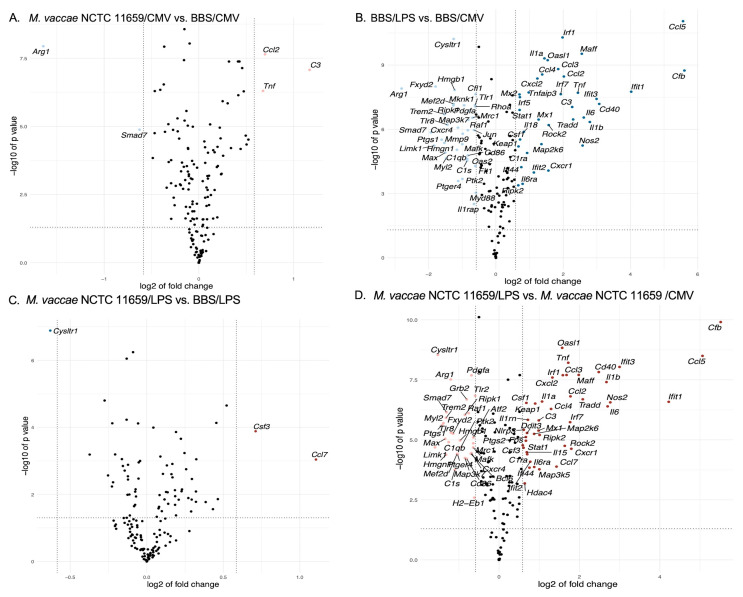
Figure illustrating volcano plots representing differentially expressed genes between pairs of treatment groups. The x-axis represents the log ratio of the fold change, and the y-axis represents the negative log of the *p*-value, which was derived from a moderated *t*-test. We used the limma R library [27] in order to calculate fold changes and *p*-values in ROSALIND^®^. Each dot in each of the figure panels represents a different gene. (**A**) The pale cornflower-blue dots (upper-left portion of the volcano plot) represent genes that were expressed at lower levels in the *M. vaccae* NCTC 11659/CMV-treated group relative to the BBS/CMV-treated group. The melon dots (upper right of the volcano plot) represent genes in the *M. vaccae* NCTC 11659/CMV-treated group that were expressed at higher levels relative to the BBS/CMV-treated group. (**B**) The pale-cornflower-blue dots (upper-left portion of the volcano plot) represent genes that were expressed at lower levels in the BBS/LPS-treated group relative to the BBS/CMV-treated group. The sea-blue dots (upper right of the volcano plot) represent genes in the BBS/LPS-treated group that were expressed at higher levels relative to the BBS/CMV-treated group. (**C**) The sea-blue dots (upper-left portion of the volcano plot) represent genes that were expressed at lower levels in the *M. vaccae* NCTC 11659/LPS-treated group relative to the BBS/LPS-treated group. The metallic-red dots (upper right of the volcano plot) represent genes in the *M. vaccae* NCTC 11659/LPS-treated group that were expressed at higher levels relative to the BBS/LPS-treated group. (**D**) The melon dots (upper-left portion of the volcano plot) represent genes that were expressed at lower levels in the *M. vaccae* NCTC 11659/LPS-treated group relative to the *M. vaccae* NCTC 11659/CMV-treated group. The metallic-red dots (upper right of the volcano plot) represent genes in the *M. vaccae* NCTC 11659/LPS-treated group that were expressed at higher levels relative to the *M. vaccae* NCTC 11659/CMV-treated group. Black dots in panels A-D represent genes that were not found to be differentially expressed between the groups. The black dashed vertical lines represent Log2 fold changes that exceed |0.6|, i.e., genes with a fold change of at least 1.5. The black dashed horizontal line in panels A-D indicates a *p*-value of 0.05, expressed as the –log10 *p*-value, with values derived from a moderated *t*-test. Volcano plots were generated using RStudio with ggplot2 and ggrepel packages. See Table S3 for a list of definitions of gene symbols.

Studies using *M. vaccae* NCTC 11659 in vivo have shown that, in the absence of subsequent stress exposure or LPS challenge, *M. vaccae* NCTC 11659 has an adjuvant-like effect, e.g., increasing bronchopulmonary *Il1b*, *Il6*, and *Tnf* mRNA expression 12 h to 3 days following intratracheal administration [25] and resulting in a persistent increase in the secretion of IL-6 from mesenteric lymph node cells stimulated with an anti-CD3 antibody in vitro [11] or a persistent increase in hippocampal *Il6* mRNA expression [20]. Likewise, in vitro studies have revealed that the treatment of human THP-1-derived-macrophages with *M. vaccae* NCTC 11659 increases the expression of genes involved in proinflammatory signaling, including *Il1b*, *Ccl2*, and numerous other canonical inflammatory markers [19] (Holbrook et al., 2023). In the present study, 48 h following the exposure of BV-2 cells to 100 µg/mL *M. vaccae* NCTC 11659, 5 of the 248 endogenous genes in the CodeSet (i.e., 2%) were differentially expressed (*p* < 0.05 and fold change either less than –1.5 or greater than 1.5) within the CMV groups between *M. vaccae* NCTC 11659 conditions (i.e., 100 µg/mL *M. vaccae* NCTC 11659/CMV vs. BBS/CMV) (Figure 3A). Of these five differentially expressed genes, three were upregulated (*C3*, *Ccl2*, and *Tnf*) and two were downregulated (*Arg1* and *Smad7*, which encodes Smad7. Smad7 inhibits transforming growth factor beta (TGFβ) signaling by preventing the formation of Smad2/Smad4 complexes that initiate TGFβ signaling [26]) (for a list of differentially expressed genes, see Table S1; for a list of genes that had *p*-values less than 0.05, see Table S2). Note that a large number of genes (47 genes with fold changes <1.5 but > 0; 61 genes with fold changes >–1.5 but < 0) had significantly different expression levels between groups based on *p*-values but did not meet the criterion for an absolute value that exceeded the Log2 fold change. 

### 2.4. Effects of LPS on mRNA Expression in Murine BV-2 Microglial Cells Based on Analysis Using the NanoString Platform

Previous studies have demonstrated that 250 ng/mL LPS (*Escherichia coli* 0111:B4) potently induces inflammation in BV-2 microglial cells and human THP-1-derived macrophages [19,21,28]. Twenty-four hours following the exposure of BV-2 cells to 250 ng/mL LPS, relative to CMV control conditions, 71 of the 248 endogenous inflammation-related genes in the CodeSet (i.e., 28.6%) were differentially expressed, as assessed by a moderated *t*-test (i.e., satisfied the criteria of *p* < 0.05 and fold change either less than –1.5 or greater than 1.5) (i.e., BBS/250 ng/mL LPS vs. BBS/CMV) (Figure 3B). Of these 71 differentially expressed genes, 36 were upregulated and 35 were downregulated (for a list of differentially expressed genes, see Table S4, and for a list of genes that had *p*-values less than 0.05, see Table S5). Note that a large number of genes had significantly different expression levels between groups based on *p*-values but did not meet the criterion for an absolute value that exceeded the Log2 fold change.

### 2.5. Differential Gene Expression between M. vaccae NCTC 11659/LPS as Compared to BBS/LPS Based on Analysis Using the NanoString Platform

Previous studies have shown that 100 µg/0.1 mL *M. vaccae* NCTC 11659, given by s.c. injection once a week for three weeks, can attenuate the stress-induced exaggeration of inflammatory hippocampal gene expression [20]. In THP-1-derived macrophages, pretreatment with 300 µg/mL *M. vaccae* NCTC 11659 24 h prior to LPS exposure, relative to the BBS/LPS condition, increases the expression of a number of genes involved in anti-inflammatory signaling, including *Il10*, *Il10rb*, *Tgfb2*, *Tgfbr1*, and *Mrc1*, and decreases the expression of genes encoding proinflammatory cytokines, such as *Il12b* [19]. In the present study, among cells exposed to LPS-challenge conditions, exposure to 100 µg/mL *M. vaccae* NCTC 11659 relative to the BBS control condition (i.e., 100 µg/mL *M. vaccae* NCTC 11659/250 ng/mL LPS vs. BBS/250 ng/mL LPS) led to the differential expression of 3 of the 248 genes in the CodeSet (i.e., 1.2%) (*p* < 0.05 and fold change either less than –1.5 or greater than 1.5) when assessed 48 h following exposure to *M. vaccae* NCTC 11659 (Figure 3C). These three differentially expressed genes (in order of descending absolute fold change) were *Ccl7* (upregulated), *Csf3* (upregulated), and *Cysltr1* (downregulated; for a list of differentially expressed genes, see Table S6; for a list of genes that had *p*-values less than 0.05, see Table S7). Note that a large number of genes had significantly different expression levels between groups based on *p*-values but did not meet the criteria for an absolute value that exceeded the Log2 fold change. 

### 2.6. Differential Gene Expression between M. vaccae NCTC 11659/LPS as Compared to M. vaccae NCTC 11659/CMV Based on Analysis Using the NanoString Platform

The NanoString panel that was used includes genes (such as *Il1b*, *Il6*, *Tnf*, *Nos2*, C-C chemokine ligand *Ccl2*, *Tlr2*, *Tgfb*, *Il10*, *Il4*, etc.) that can provide insight into whether or not the murine BV-2 microglial cells are polarized toward a proinflammatory phenotype or polarized toward an anti-inflammatory phenotype [29,30]. Among *M. vaccae* NCTC 11659-treated cells, 24 h of exposure to 250 ng/mL LPS, relative to the CMV control condition (i.e., 100 µg/mL *M. vaccae* NCTC 11659/250 ng/mL LPS vs. 100 µg/mL *M. vaccae* NCTC 11659/CMV), led to the differential expression of 72 of the 248 genes in the CodeSet (i.e., 29.03%) (*p* < 0.05 and fold change either less than –1.5 or greater than 1.5) (Figure 3D) (for a list of differentially expressed genes, see Table S8; for a list of genes that had *p*-values less than 0.05, see Table S9). Note that a large number of genes had significantly different expression levels between groups based on *p*-values but did not meet the criterion for an absolute value that exceeded the Log2 fold change. Despite preexposure to *M. vaccae* NCTC 11659, LPS challenge, relative to the CMV control condition, increased the markers of M1 polarization, including *Il1b*, *Il6*, *Tnf*, *Nos2*, and C-C chemokine ligand *Ccl2*, while it decreased *Arg1*, a marker of M2 polarization, suggesting that *M. vaccae* NCTC 11659 was not sufficient to prevent an LPS-induced shift toward an M1 microglia phenotype.

### 2.7. Validation of Effects of M. vaccae NCTC 11659 and LPS on Arg1 Gene Expression in BV-2 Microglial Cells Using Real-Time RT-PCR

**Figure 4:**
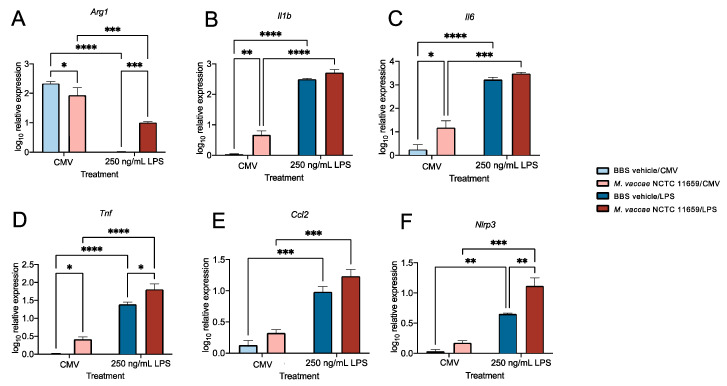
Pretreatment with *M. vaccae* NCTC 11659 attenuated lipopolysaccharide (LPS)-induced suppression of *Arg1* expression while inducing microglial priming. Expression of (**A**) *Arg1*, (**B**) *Il1b*, (**C**) *Il6*, (**D**) *Tnf*, (**E**) *Ccl2*, and (**F**) *Nlrp3* was measured using real-time reverse transcription polymerase chain reaction (real-time RT-PCR) and is represented relative to the highest Ct value for each gene using the 2^−ΔΔCt^ method. Data in panels A-F represent means + SEM. Data were analyzed using a two-factor ANOVA followed by Fisher’s LSD test using a pooled error value, if appropriate, and a two-tailed alpha level of 0.05. * *p* < 0.05, ** *p* < 0.01, *** *p* < 0.001, **** *p* < 0.0001. For all experimental conditions, there were 3 technical replicates (*n* = 3) from *n* = 1 experiment. Abbreviations: BBS, borate-buffered saline; CMV, cell media vehicle; LPS, lipopolysaccharide; NCTC, National Collection of Type Cultures. For definitions of gene symbols, see Table S3. For sample sizes, see Table S10.

The analysis using the NanoString platform indicated the decreased expression of *Arg1* in murine BV-2 microglial cells following exposure to either *M. vaccae* NCTC 11659 or LPS (Figure 3A,B). To validate these *Arg1* gene expression results, *Arg1* gene expression was analyzed using real-time RT-PCR, using *Actb* as a housekeeping gene. We analyzed *Actb* for differential expression across treatment groups and found no differences in expression (Figure S2). The analysis of *Arg1* expression revealed an interaction between *M. vaccae* NCTC 11659 and LPS (*F*_(1,6)_ = 40.70, *p* < 0.001; Figure 4A, Tables S10 and S11). The post hoc analysis revealed a decrease in *Arg1* expression in the *M. vaccae* NCTC 11659/CMV group relative to the BBS/CMV control condition (*p* < 0.05), as well as decreased *Arg1* expression in the BBS/250 ng/mL LPS group relative to the BBS/CMV vehicle condition (*p* < 0.0001). Although not detected based on the analysis using the NanoString platform, among the LPS-challenged groups, exposure to *M. vaccae* NCTC 11659 increased *Arg1* expression (*p* < 0.001). Among *M. vaccae* NCTC 11659-exposed cells, LPS induced a significant, but attenuated, decrease in *Arg1* expression (*p* < 0.001) (Figure 4A). It is important to note that, although *M. vaccae* NCTC 11659 attenuated the LPS-induced decrease in *Arg1* mRNA expression, it did not prevent it.

### 2.8. Validation of Effects of M. vaccae NCTC 11659 and LPS on Genes Encoding Proinflammatory Cytokines and Chemokine Ligands (i.e., Il1b, Il6, Tnf, and Ccl2 Gene Expression) in BV-2 Microglial Cells Using Real-Time RT-PCR

The analysis using the NanoString platform revealed that exposure to *M. vaccae* NCTC 11659 alone increased the expression of *Tnf* and *Ccl2*, while LPS alone or in the presence of *M. vaccae* NCTC 11659 increased the expression of *Il1b*, *Il6*, *Tnf*, and *Ccl2* in BV-2 microglial cells (Figure 3A,B,D). To validate these results from the NanoString analysis, *Il1b*, *Il6*, *Tnf*, and *Ccl2* gene expression levels were analyzed using real-time RT-PCR, using *Actb* as a housekeeping gene.

The analysis of *Il1b* mRNA expression revealed an interaction between *M. vaccae* NCTC 11659 and LPS (*F*_(1,6)_ = 6.238, *p* < 0.05; Figure 4B and Table S10). The post hoc analysis revealed increased *Il1b* expression in the *M. vaccae* NCTC 11659/CMV group relative to the BBS/CMV control condition (*p* < 0.01) (Figure 4B and Figure S3). It was determined that the BBS/250 ng/mL LPS group had increased *Il1b* mRNA expression relative to the BBS/CMV vehicle condition (Figure 4B; *p* < 0.0001) (Figure S4). Among *M. vaccae* NCTC 11659-treated cells, LPS still induced a significant increase in *Il1b* mRNA expression (*p* < 0.0001) (Figure 4B and Figure S6, Table S10).

The analysis of *Il6* mRNA expression revealed main effects for both *M. vaccae* NCTC 11659 (*F*_(1,6)_ = 10.6, *p* < 0.05; Figure 4C and Table S10) and LPS (*F*_(1,6)_ = 210.5, *p* < 0.0001; Figure 4C and Table S10). The post hoc analysis revealed increased *Il6* mRNA expression in the *M. vaccae* NCTC 11659/CMV group relative to the BBS/CMV control condition (*p* < 0.05) (Figure 4C and Figure S3). Unsurprisingly, the relative expression of *Il6* mRNA was higher in the BBS/250 ng/mL LPS group relative to the BBS/CMV vehicle condition (*p* < 0.0001) (Figure 4C and Figure S4). Among *M. vaccae* NCTC 11659-treated cells, LPS still induced a significant increase in *Il6* mRNA expression (*p* < 0.001) (Figure 4C and Figure S6, Table S10). 

The analysis of *Tnf* mRNA expression revealed main effects of both *M. vaccae* NCTC 11659 (*F*_(1,6)_ = 14.88, *p* < 0.01; Figure 4D and Table S10) and LPS (*F*_(1,6)_ = 169.2, *p* < 0.0001; Figure 4D and Table S10). The post hoc analysis revealed that *M. vaccae* NCTC 11659 alone increased *Tnf* mRNA expression (i.e., *M. vaccae* NCTC 11659/CMV group relative to the BBS/CMV control condition (*p* < 0.05)) (Figure 4D and Figure S3). Likewise, LPS increased *Tnf* mRNA expression (i.e., BBS/250 ng/mL LPS group relative to the BBS/CMV vehicle condition; *p* < 0.0001) (Figure 4D and Figure S4). Among the LPS-challenged groups, exposure to *M. vaccae* NCTC 11659 increased *Tnf* mRNA expression (*p* < 0.05) (Figure 4D and Figure S5, Table S10). Among the *M. vaccae* NCTC 11659 groups, exposure to LPS increased *Tnf* mRNA expression (*p* < 0.0001) (Figure 4D and Figure S6).

The analysis of *Ccl2* mRNA expression revealed a main effect of LPS (*F*_(1,6)_ = 90.52, *p* < 0.001; Figure 4E and Table S10). The post hoc analysis revealed greater *Ccl2* mRNA expression in the BBS/250 ng/mL LPS group relative to the BBS/CMV vehicle condition (*p* < 0.001) (Figure 4E). Among the *M. vaccae* NCTC 11659-treated groups, those challenged with LPS had significantly greater *Ccl2* mRNA expression levels (*p* < 0.001) (Figure 4E and Table S10).

### 2.9. Validation of Effects of M. vaccae NCTC 11659 and LPS on Nlrp3 mRNA Expression in BV-2 Microglial Cells Using Real-Time RT-PCR

Our prior work suggests that *Nlrp3* inflammasome priming plays a pivotal role in stress-induced neuroinflammatory and microglial priming [31]. Conversely, immunization with whole-cell, heat-killed *M. vaccae* NCTC 11659 by itself decreases hippocampal *Nlrp3* mRNA expression and attenuates stress-induced hippocampal microglial priming when assessed one week later [13]. The analysis using the NanoString platform demonstrated an increase in the expression of *Nlrp3* mRNA following exposure to LPS among BV-2 microglial cells previously exposed to *M. vaccae* NCTC 11659 (Figure 3D). To validate these results, *Nlrp3* mRNA expression was analyzed using real-time RT-PCR, using *Actb* as a housekeeping gene. The analysis of *Nlrp3* mRNA expression revealed main effects for both *M. vaccae* NCTC 11659 (*F*_(1,6)_ = 11.63, *p* < 0.05; Figure 4F and Table S10) and LPS (*F*_(1,6)_ = 78.61, *p* < 0.0001; Figure 4F and Table S10). The post hoc analysis revealed that *Nlrp3* mRNA expression was greater in the BBS/250 ng/mL LPS group relative to the BBS/CMV vehicle condition (*p* < 0.01; Figure 4F). Among *M. vaccae* NCTC 11659-treated groups, LPS increased *Nlrp3* mRNA expression (i.e., *M. vaccae* NCTC 11659/LPS group relative to the *M. vaccae* NCTC 11659/BBS control condition (*p* < 0.001; Figure 4F)). Likewise, greater *Nlrp3* mRNA expression was observed in the *M. vaccae* NCTC 11659/250 ng/mL LPS group relative to the BBS/250 ng/mL LPS vehicle condition (*p* < 0.01) (Figure 4F and Table S10), suggesting that *M. vaccae* NCTC 11659 promoted neuroinflammatory and microglial priming. For access to all data for all studies reported here, see File S1.

## 3. Discussion

Here, we report that the exposure of murine BV-2 microglial cells to *M. vaccae* NCTC 11659 by itself induced an “adjuvant-like” effect, increasing the expression of canonical proinflammatory genes, such as *Il1b*, *Il6*, and *Tnf*. As expected, the exposure of murine BV-2 microglial cells to LPS induced a strong polarization of BV-2 microglia toward an inflammatory phenotype relative to the CMV vehicle control condition, including increases in the expression of genes encoding canonical proinflammatory cytokines, chemokine ligands, and the Nlrp3 inflammasome. In most cases, pretreatment with *M. vaccae* NCTC 11659 was found to enhance the effects of LPS, suggesting that *M. vaccae* NCTC 11659 exaggerated the polarization of BV-2 cells, inducing neuroinflammatory and microglial priming responses. However, treatment with *M. vaccae* NCTC 11659 protected against the LPS-induced reduction in *Arg1*, suggesting that *M. vaccae* NCTC 11659 attenuated the LPS-induced shift toward an inflammatory metabolic phenotype [29,30,32,33,34,35]. Together, these data suggest that exposure to *M. vaccae* NCTC 11659 induces an atypical microglial phenotype, characterized by enhanced markers of neuroinflammation and microglial priming, while at the same time attenuating the LPS-induced decrease in *Arg1*, suggesting an attenuation of the LPS-induced inflammatory metabolic phenotype.

### 3.1. M. vaccae NCTC 11659 by Itself Polarized Murine BV-2 Microglial Cells toward a Proinflammatory Phenotype

Treatment with *M. vaccae* NCTC 11659 alone induced an “adjuvant-like” effect in BV-2 microglial cells, which, based on real-time RT-PCR, was evidenced by the increase in the gene expression of *Il1b*, *Il6*, and *Tnf.* This was expected as, when animals are immunized with *M. vaccae* NCTC 11659 without subsequent exposure to a stressor, *M. vaccae* NCTC 11659 induces small fold-change increases in markers of inflammation. For example, the intratracheal administration of *M. vaccae* NCTC 11659 in s.c. *M. vaccae* NCTC 11659-immunized mice (days –28 and –14) induces increases in bronchopulmonary *Il1b*, *Il6*, and *Tnf* mRNA expression 12 h to 3 days later [25]. Similarly, the s.c. administration of *M. vaccae* NCTC 11659 in unstressed, single-housed control mice increases the secretion of IL-6 from freshly isolated mesenteric lymph node cells stimulated with an anti-CD3 antibody, assessed three weeks following immunization [11]. Finally, the s.c. administration of *M. vaccae* NCTC 11659 increases hippocampal *Il6* mRNA expression in unstressed control rats, assessed one week following immunization [20]. Consistent with these significant, but small fold-change, increases in proinflammatory gene expression, 47 genes in the CodeSet were significantly increased, as assessed at *p* < 0.05, but did not exceed the threshold of 1.5-fold change conventionally required for differential expression. 

### 3.2. LPS Strongly Polarizes Murine BV-2 Microglial Cells toward a Proinflammatory Phenotype

The exposure of murine BV-2 microglial cells to LPS, as expected, induced a strong polarization toward a proinflammatory phenotype when assessed 24 h later, with the differential expression of 28.6% of the genes analyzed relative to the CMV control condition, including increases in the expression of genes encoding canonical proinflammatory cytokines such as IL-1β, IL-6, and Tnf, and chemokine ligands such as Ccl2. The induction of inflammatory mRNA expression by LPS in murine BV-2 cells is consistent with previous studies [23,36,37,38]. Conversely, LPS decreased the expression of *Arg1* and *Mrc1* (*Cd206*, encoding mannose receptor C-Type 1), likewise consistent with the polarization of murine BV-2 microglial cells toward a more inflammatory phenotype [29,35].

### 3.3. M. vaccae NCTC 11659 Enhanced the Polarizing Effects of LPS on Inflammatory mRNA Expression in Murine BV-2 Microglial Cells, Consistent with Neuroinflammatory and Microglial Priming

Among cells exposed to LPS, exposure to *M. vaccae* NCTC 11659 primed murine BV-2 microglial cells to respond to LPS with exaggerated increases in the expression of canonical inflammatory markers, i.e., *Tnf* and *Nlrp3*, which plays a pivotal role in stress-induced neuroinflammatory and microglial priming [31]. These effects were confirmed using real-time RT-PCR. The overall effects of *M. vaccae* NCTC 11659 in enhancing inflammatory responses induced by LPS in microglia are distinct from the effects of *M. vaccae* NCTC 11659 on peripheral immune cells. Previous studies have shown that human THP-1-derived macrophages respond to exposure to *M. vaccae* NCTC 11659 with attenuated LPS-induced decreases in *Il10*:*Il12b*, *Il10*:*Il23a*, *Tgfb1*:*Il12b*, and *Tgfb1*:*Il23a* mRNA expression ratios [19]. 

Previous studies conducted in vivo demonstrate that the peripheral administration (i.e., s.c.) of *M. vaccae* NCTC 11659 attenuates stressor-induced inflammation, neuroinflammation, and microglial priming while promoting stress resilience [6,11,12,13,20]. The current study implies that the ability of *M. vaccae* NCTC 11659 to attenuate stressor-induced neuroinflammation and microglial priming is not due to direct exposure to the residential immune cells in the CNS but rather through the peripheral immune system, secondarily impacting microglial cells. Another possibility is that the anti-inflammatory effects of *M. vaccae* NCTC 11659 emerge over a longer time frame. Studies conducted using immunization with *M. vaccae* NCTC 11659 tend to evaluate impacts on stress-induced inflammatory responses at least one week following immunization, whereas the studies conducted here evaluated responses to LPS one day following treatment with *M. vaccae* NCTC 11659 [6,11,12,13,20]. Indeed, increases in the serum concentrations of anti-inflammatory cytokines, such as IL-4, IL-10, and TGFβ, in mice peak four to six weeks following infection with a strain of *M. vaccae* isolated from bovine submaxillary lymph nodes [39], suggesting a delayed and persistent anti-inflammatory response to *M. vaccae* in vivo.

### 3.4. M. vaccae NCTC 11659 Attenuated LPS-Induced Reduction in Arg1 Gene Expression

The dramatic LPS-induced reduction in *Arg1* was blunted by previous exposure to *M. vaccae* NCTC 11659. These data suggest that the direct exposure of BV-2 microglia to *M. vaccae* NCTC 11659 induces a complex microglial phenotype characterized by increased neuroinflammation and microglial priming, in association with the attenuation of LPS-induced decreases in *Arg1*. Since Arg1 is characteristic of microglia involved in wound healing, the phagocytosis of debris, inflammation inhibition, homeostasis restoration, and extracellular matrix protection [35], this finding raises the question of whether *M. vaccae* NCTC 11659 might, at the same time, (1) induce microglial priming and (2) promote a metabolic phenotype consistent with recovery from tissue damage. Future studies should evaluate the time course of gene expression in the current model to determine whether *M. vaccae* NCTC 11659 accelerates the transition to an anti-inflammatory phenotype following the initial immune activation.

### 3.5. Comparisons of the Effects of M. vaccae NCTC 11659 on the Immunophenotype of Microglia Following Administration In Vivo versus In Vitro

Previous studies have shown that when *M. vaccae* NCTC 11659 is given in vivo by subcutaneous injection, it can prevent microglial priming in rats when assessed one week later [12,13,14]. Interestingly, when given in vivo in rats, *M. vaccae* NCTC 11659 decreases hippocampal *Nfkbia* [12,13] and *Nlrp3* [13] mRNA expression, consistent with the finding that *M. vaccae* NCTC 11659 can prevent stress-induced hippocampal neuroinflammation and the priming of hippocampal microglia [12,13,14,20] when assessed one week after subcutaneous administration. In contrast, *M. vaccae* NCTC 11659 appears to have starkly different effects when applied directly to BV-2 cells, a hippocampal microglial cell line, when assessed 48 h following exposure. Specifically, under LPS-challenge conditions, the direct preexposure of BV-2 cells to *M. vaccae* NCTC 11659 enhanced LPS-induced increases in *Nlrp3* mRNA expression, suggesting that either (1) the ability of the in vivo administration of *M. vaccae* NCTC 11659 to decrease *Nlrp3* mRNA expression is not due to the direct effects of the bacterium on microglial cells or (2) the ability of *M. vaccae* NCTC 11659 to decrease *Nlrp3* mRNA expression in the CNS occurs over a longer time course. One exception to these differences in the effects of *M. vaccae* NCTC 11659 when administered in vivo versus in vitro is the effect on *Arg1* mRNA expression, a marker of an M2-like immunophenotype. *M. vaccae* NCTC 11659 increases *Arg1* mRNA expression when administered either in vivo [12] or, specifically under LPS-challenge conditions, in vitro, suggesting that this is a highly conserved effect of *M. vaccae* NCTC 11659 on microglial cells. Since *Arg1* expression in microglia is characteristic of an M2 anti-inflammatory phenotype [40,41], this may represent a conserved bias toward an atypical M2-like microglia phenotype in response to *M. vaccae* NCTC 11659 both in vivo and in vitro. This may be beneficial in some conditions, such as neurodegenerative diseases [40,42,43] and psychiatric disorders [30].

### 3.6. Limitations

One limitation of this report is that this study was conducted in vitro. It will be important to determine whether whole-cell, heat-killed *M. vaccae* NCTC 11659, when administered in vivo by either s.c. injection, intradermal injection, or other routes (e.g., oral, intranasal, intragastric, intratracheal, and intravenous routes), accesses the CNS, as observed with *M. tuberculosis* [44]. Alternatively, *M. vaccae* NCTC 11659 may be phagocytosed by circulating monocytes or dendritic cells, which can enter the circulation and translocate across the blood–cerebrospinal fluid barrier or blood–brain barrier [44,45,46], in which case, it may have very different effects on microglial phenotypes. In addition, it will be important to fully characterize the time course of the effects of *M. vaccae* NCTC 11659 on inflammatory responses to a subsequent immune challenge beyond the 48 h time point studied here. Although we have characterized the immunophenotype of BV-2 cells in response to *M. vaccae* NCTC 11659 and LPS, the effects of treatment on the external morphology of the cells are also an important concern and should be addressed in future studies. Another point to consider is that the BV-2 cell line is derived from female mice. It will be important to see whether primary microglial cells, including microglial cells extracted from male mice, have a similar response to *M. vaccae* NCTC 11659. 

### 3.7. Clinical Implications

The data from the current study suggest that the ability of immunization with *M. vaccae* NCTC 11659 or its component parts in vivo to attenuate LPS-induced inflammatory responses in microglia, as observed previously [12,13], appears to be due to the regulation of the peripheral immune system rather than due to direct actions on microglial cells. However, future studies of murine BV-2 microglia with extended time courses are required to determine whether the anti-inflammatory effects of *M. vaccae* NCTC 11659 are evident at time points when the anti-inflammatory effects of *M. vaccae* NCTC 11659 are observed in vivo, typically one to two weeks following treatment. In addition, future studies using primary cultures of microglia are needed to confirm that BV-2 cells and freshly isolated microglia respond to *M. vaccae* NCTC 11659 in a similar way. Nevertheless, the ability of *M. vaccae* NCTC 11659 to increase *Arg1* mRNA expression, as observed now both in vivo [12] and in vitro, may lead to a less aggressive M1-like inflammatory environment, which may be beneficial in some conditions, including neurodegenerative diseases [40,42,43] and psychiatric disorders [30].

### 3.8. Conclusions and Future Directions

Although *M. vaccae* NCTC 11659 has been studied primarily in the context of the “Old Friends” hypothesis, the current study highlights how whole-cell, heat-killed *M. vaccae* NCTC 11659 acutely (i.e., within 48 h) enhanced LPS-induced inflammation in murine BV-2 cells, suggestive of enhanced neuroinflammation and microglial priming. These data indicate that the ability of *M. vaccae* NCTC 11659, when administered in vivo, to prevent stress-induced microglial priming in association with stress resilience effects in rodent models either is due to *M. vaccae* NCTC 11659’s effects on the peripheral immune system, which indirectly attenuates stress-induced neuroinflammation and microglial priming, or evolves over a longer time course, or is due to a combination of these factors. In support of the hypothesis that the strong anti-inflammatory and immunoregulatory effects of *M. vaccae* NCTC 11659 develop over a longer time course, early responses to the administration of *M. vaccae* NCTC 11659 in vivo are dominated by proinflammatory responses (i.e., increased *Il1b*, *Il6*, and *Tnf* mRNA expression 12–72 h following administration) [25], whereas the anti-inflammatory and immunoregulatory effects of *M. vaccae* NCTC 11659 are typically observed later, i.e., one week after administration [11,12,13,14,20]. Future studies are needed to determine whether *M. vaccae* NCTC 11659 or its component parts can access the CNS compartment, either as a single bacillus or in a “Trojan horse”-like manner, as has been observed with *M. tuberculosis*. 

## 4. Materials and Methods

### 4.1. Murine BV-2 Microglial Cells

BV-2 microglial cells are an immortalized murine cell line that has been infected with a *v-raf/v-myc* oncogene-carrying retrovirus (J2) [47]. We selected murine BV-2 microglial cells to evaluate the effects of *M. vaccae* NCTC 11659 on naïve and LPS-stimulated immune responses, as this cell line reproduces the effects of LPS on primary microglia with high fidelity while reducing the need for continuous cell preparation and animal experimentation [48]. Cells were obtained from frozen stocks maintained in Gibco^TM^ Recovery™ Cell Culture Freezing Medium (Cat. No. 12648-010, Thermo Fisher Scientific, Waltham, MA, USA). Briefly, for freezing cells, cells were cultured to be approximately 70–80% confluent, viable, and healthy before freezing. Fresh medium was added 5 h before freezing. For a 100 mm culture plate, 1.5–2 mL of trypsin plus 5 mL of medium was added to neutralize the trypsin. Cells were centrifuged at 1500 r.p.m. for 5 min, and the supernatant was discarded. Cells were resuspended in Gibco^TM^ Recovery™ Cell Culture Freezing Medium and gently mixed to maintain a homogeneous cell suspension. Aliquots of 1 mL of cell suspension were dispensed into sterile cryogenic storage vials. Cryovials containing the cells were placed in an isopropanol chamber and stored at −80 °C overnight. The next day, vials containing frozen cells were transferred to liquid nitrogen.

Cells were received on passage four. Because they were semi-suspended, BV-2 cells were shaken to be lifted during passage. Cells were not used for more than 20 passages. For the experiments described here, BV-2 cells were cultured in Gibco^TM^ Advanced DMEM/F12 (Cat. No. 12634010; Thermo Fisher Scientific) medium supplemented with Hyclone 10% fetal bovine serum (FBS; Cat. No. F9423, Sigma-Aldrich, Saint Louis, MO, USA) and Gibco^TM^ 1% penicillin/streptomycin (100 U/mL penicillin and 100 μg/mL streptomycin; Cat. No. 15140122, Thermo Fisher Scientific) under standard culture conditions (37 °C in a humidified 5% CO_2_ incubator). The cells were plated on sterile 24-well tissue culture plates (Cat. No. 10062-896; VWR North American, Radnor, PA, USA). Each well was plated with 200,000 BV-2 cells in 0.5 mL of medium. To harvest BV-2 cells, the cells were washed with PBS prior to the addition of Gibco^TM^ Trypsin-EDTA (0.25%) (Cat. No. 25200056, Thermo Fisher Scientific); the cells were then centrifuged (500 r.p.m. for 5 min at 21–23 °C) and resuspended in DMEM/F12 medium.

### 4.2. M. vaccae NCTC 11659

The material used here was provided as a 10 mg/mL stock suspension in sterile borate-buffered saline; strain National Collection of Type Cultures (NCTC) 11659, batch C079-ENG#1, provided by BioElpida (Lyon, France). For the heat-killing of *M. vaccae* NCTC 11659, the culture was centrifuged at 3000× *g* at 4 °C for ten minutes to pellet the cells, the growth medium was removed, and cells were weighed and resuspended in sterile borate-buffered saline (BBS) to a concentration of 10 mg/mL. Cells were transferred to a sealed sterile glass container and autoclaved at 121 °C for 15 min. The sterile heat-killed bacterial stock was stored at 4 °C, and *M. vaccae* NCTC 11659 was further diluted to a final in-well concentration of 96.99 μg/mL for the treatment of BV-2 cells. Stocks were swirled each time before they were pipetted into the wells to ensure that the suspension was distributed equally. After pipetting *M. vaccae* NCTC 11659 or vehicle into the wells, the plates were gently swirled to evenly distribute the treatments. For quality control, the *M. vaccae* NCTC 11659 material was tested to confirm its immunoregulatory effects (e.g., increased *Il10*:*Il12a* and *Il10*:*Il12b* mRNA expression) in murine bone-marrow-derived dendritic cells. 

### 4.3. LPS

This study used lipopolysaccharide (LPS; *Escherichia coli* O111:B4; Cat. No. L2630, Sigma-Aldrich) as an immune challenge, while the remaining wells were challenged with a cell culture media vehicle (CMV) control condition. It is well documented that LPS (*Escherichia coli* O111:B4) induces a proinflammatory response, identified by canonical proinflammatory markers, in BV-2 microglial cells [23,36,37].

### 4.4. Experimental Timeline

**Figure 5:**
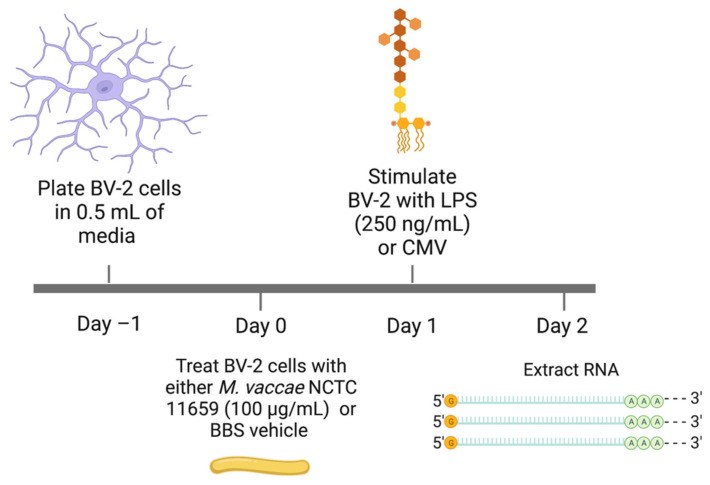
Experimental timeline. Abbreviations: BBS, borate-buffered saline; CMV, cell media vehicle; LPS, lipopolysaccharide (*E. coli* O111:B4); NCTC, National Collection of Type Cultures; RNA, ribonucleic acid.

The experimental timeline is illustrated in Figure 5. The experiment was conducted once, and the statistical analyses were performed on technical replicates. On Day 0, we treated six wells (200,000 BV-2 cells in 0.5 mL of medium) with a whole-cell, heat-killed preparation of *M. vaccae* NCTC 11659 (15 μL of 3.33 mg/mL *M. vaccae* NCTC 11659 in a final volume of 515 μL per well, resulting in a final concentration of 96.99 µg/mL, hereafter referred to as 100 µg/mL, of *M. vaccae* NCTC 11659) and six wells with 15 μL of vehicle (sterile BBS). Twenty-four hours later, on Day 1, we challenged three of the *M. vaccae* NCTC 11659-treated wells and three of the BBS-treated wells with 10 µL of 13.25 µg/mL LPS stock, resulting in each well having a concentration of 252.4 ng/mL LPS, hereafter referred to as 250 ng/mL (LPS; *Escherichia coli* O111:B4; Cat. No. L2630, Sigma-Aldrich), while the remaining wells were challenged with a cell culture media vehicle (CMV) control condition. On Day 2, the collection of BV-2 cells and the purification of total RNA were carried out, as described in Section 4.5.

### 4.5. RNA Isolation

Twenty-four hours after treating the cells with 250 ng/mL LPS (*E. coli* O111:B4) or CMV, total RNA was extracted from BV-2 cells using the QIAGEN RNeasy Mini kit (Cat. No. 74104, QIAGEN, Hilden, Germany). For each well, RNA extraction yielded >30 μg of RNA. Following the determination of RNA concentrations in each sample using a NanoDrop One machine (Cat. No. ND-ONE-W, Thermo Fisher Scientific), the RNA samples were diluted using nuclease-free water to 5 ng/µL. RNA samples were frozen at –80 °C before submitting samples to the Veterans Health Administration, Rocky Mountain Regional Veterans Affairs Medical Center (RMRVAMC) Core Equipment facility.

### 4.6. NanoString nCounter Gene Expression

An analysis of NanoString nCounter gene expression was conducted according to vendor instructions. Briefly, 25 ng of total RNA per sample was processed with the NanoString nCounter system (NanoString, Seattle, WA, USA) using the nCounter Mouse Inflammation v2 Panel (Cat. No. XT-CSO-MIN2-12; NanoString). The NanoString nCounter^®^ Mouse Inflammation v2 Panel is a multiplex gene expression analysis platform that enables the analysis of 254 genes representing a range of inflammation-related pathways. The CodeSet includes 248 murine inflammation-related genes and 6 genes designated as housekeeping genes. A number of published studies have used the nCounter Mouse Inflammation v2 Panel [49,50,51,52,53,54,55,56,57,58,59,60,61,62,63,64,65,66,67,68,69,70,71,72,73,74,75,76]; to the best of our knowledge, however, this is the first study to use this platform to study murine BV-2 cells.

The 25 ng samples of RNA were mixed with the Reporter Codeset and Capture Probeset and then incubated at 65 °C for at least 16 h to allow adequate hybridization. Hybridization buffer was added to bring the samples to a volume of 30 µL, which was then loaded into the cartridge and run on the nCounter Sprint profiler. The data were normalized through RUV-III [77]. The normalized data were then exported into ROSALIND^®^ (San Diego, CA, USA), a cloud-based genomics and gene expression analysis platform, and were analyzed using OnRamp software (version 3.35.12.0, OnRamp Bioinformatics, Inc., San Diego, CA, USA).

### 4.7. Real-Time RT-PCR and Primers

Real-time RT-PCR was used to analyze gene expression in two separate studies: Experiment 1, which evaluated the dose-dependent effects of LPS, and Experiment 2, which evaluated the effects of *M. vaccae* NCTC 11659, LPS, and their interaction. The methods used to conduct real-time RT-PCR for Experiment 1 and Experiment 2 were identical.

Aliquots of the stock samples of extracted mRNA used for the analysis using NanoString in Experiment 2 were also used for cDNA synthesis in order to perform real-time RT-PCR. For Experiments 1 and 2, approximately 500 ng of total RNA was used for the preparation of cDNA, which was conducted using Invitrogen SuperScript^TM^ II Reverse Transcriptase (Cat. No. 18064014, Thermo Fisher Scientific) according to manufacturer instructions. Approximately 50 ng per well of cDNA was used as a template material for conducting real-time RT-PCR. Real-time RT-PCR was carried out in duplicate using a CFX96 Touch Real-Time PCR Detection System (Cat. No. 1845097, Bio-Rad, Hercules, CA, USA), together with QuantiTect SYBR Green Master Mix (Cat. No. 204145, QIAGEN, Hilden, Germany). For both Experiments 1 and 2, gene expression was normalized using *Actb*, which encodes beta-actin. Real-time RT-PCR data were represented as a fold increase relative to the lowest amount of mRNA expressed on the plate for each gene using the 2^–∆∆Ct^ method.

Primer sequences (Table 1) other than *Arg1* were designed using the PrimerQuest^TM^ Tool (Integrated DNA Technologies (IDT) website; https://www.idtdna.com/pages, accessed on 8 August 2019, Coralville, IA, USA). Primer sequences were designed to span exon/exon boundaries in order to exclude the amplification of genomic DNA. The murine *Arg1* primer sequence (Table 1) was obtained from PrimerBank (https://pga.mgh.harvard.edu/primerbank/, accessed on 28 December 2023, Cambridge, MA, USA). Sequence specificity was tested using the Basic Local Alignment Search Tool at NCBI [77]. Primers were obtained from IDT. Primer specificity was verified by melt curve analysis.

### 4.8. Statistical Analysis

Statistical approaches to the analysis of NanoString data and real-time RT-PCR data are outlined in detail below.

#### 4.8.1. NanoString Analysis

The analysis of reference genes using the NanoString platform revealed that treatment conditions altered the expression of reference genes in the nCounter Mouse Inflammation v2 Panel, resulting in inadequate normalization (Figure S1). Therefore, we conducted the normalization of NanoString data in the statistical software program R using Removing Unwanted Variation-III (RUV-III), which selects stably expressed endogenous genes to use as housekeeping genes post hoc [78].

RUV-III-normalized data were imported into ROSALIND^®^. Normalized data were analyzed using ROSALIND^®^ (https://rosalind.bio/, accessed on 1 October, 2023), with a HyperScale architecture developed by ROSALIND^®^, Inc. The limma R library [27] was used to calculate fold changes and *p*-values in ROSALIND^®^ using a moderated *t*-test. The moderated *t*-test is often used for the analysis of studies with small sample sizes and can be interpreted in a similar manner to Student’s *t*-test, but with the noteworthy difference that the standard errors are moderated using empirical Bayes methods [79,80]. The statistical software program R was used to make principal coordinate analysis (PCoA) plots using the ggplot2, vegan, and ecodist packages. The statistical software program R was used to make volcano plots using the ggplot2 and ggrepel packages. Scatterplots were made using the ggplot2 and ggrepel R packages using the Log base 2 fold changes and *p*-values obtained from ROSALIND^®^.

#### 4.8.2. Real-Time RT-PCR Analysis

Relative gene expression was calculated using the 2^−ΔΔCt^ method [81], and relative gene expression data were analyzed using a two-way ANOVA followed by Fisher’s LSD test, if appropriate, at a two-tailed alpha level of 0.05 using a single pooled error term for Fisher’s LSD test [82].

## Figures and Tables

**Table 1:** Primers used for real-time RT-PCR. Primers are listed from 5′ to 3′.

Gene	Forward Primer	Reverse Primer	Sequence Name
*Actb*	TCGTGCGTGACATCAAAGAG	GGATTCCATACCCAAGAAGG	β-Actin, cytoskeletal protein (housekeeping gene)
*Arg1*	TGTCCCTAATGACAGCTCCTT	GCATCCACCCAAATGACACAT	Arginase 1
*Ccl2*	GGCTCAGCCAGATGCAGTTAA	CTTGGTGACAAAAACTACAGCTTC	C-C motif chemokine ligand 2
*Il1b*	TGGCAACTGTTCCTGAACTTC	GGAAGCAGCCCTTCATCTTT	Interleukin 1 beta
*Il6*	GAAAAGAGTTGTGCAATG	TATGGTACTCCAGAAGAC	Interleukin 6
*Il10*	GGACTTTAAGGGTTACTTGG	TCACCCAGGGAATTCAAATG	Interleukin 10
*Nfkb1*	GGATGACAGAGGCGTGTATTAG	CCTTCTCTCTGTCTGTGAGTTG	Nuclear factor of kappa light polypeptide gene enhancer in B cells 1, p105
*Nlrp3*	GAGCCTACAGTTGGGTGAA	CCTACCAGGAAATCTCGAAGAC	NLR family pyrin domain containing 3
*Tnf*	CCCTCACACTCAGATCATCT	TGTCTTTGAGATCCATGCCG	Tumor necrosis factor

## Data Availability

All data presented are available in this article and the Supplementary Materials.

## Supplementary Materials

The following supporting information can be downloaded at https://www.mdpi.com/article/10.3390/ijms25010474/s1.
