# Supplementary material for: Effects of Mycobacterium vaccae NCTC 11659 and Lipopolysaccharide Challenge on Polarization of Murine BV-2 Microglial Cells
Source: Int J Mol Sci. 2023 Dec 29;25(1):474. doi: 10.3390/ijms25010474 (PMC10779110; doi:10.3390/ijms25010474)
Supplement: Supplementary file 1 [file ijms-25-00474-s001.zip › ijms-2721985-supplementary_latest.pdf]

Supplemental materials

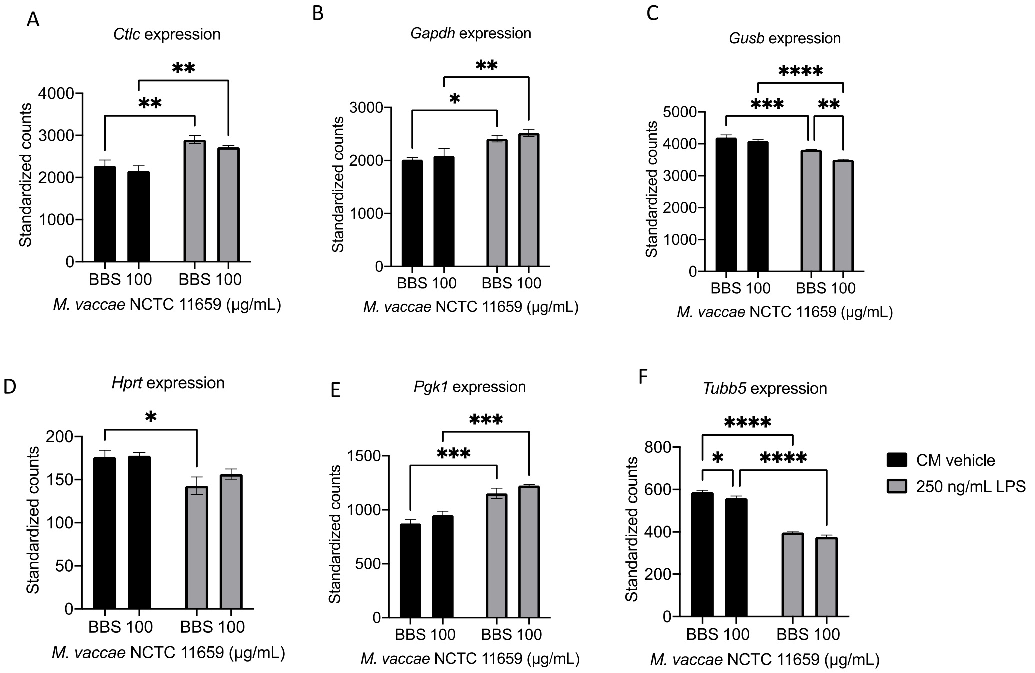

Figure S1. Treating murine BV-2 microglial cells with *M. vaccae* NCTC 11659 (100 µg/mL, 48 h) or sterile borate-buffered saline (BBS) vehicle control conditions and lipopolysaccharide (LPS; 250 ng/mL, 24 h) or a cell culture media (CM) vehicle control condition resulted in differential expression of all internal housekeeping genes in the nCounter Mouse Inflammation v2 Panel. Graphs illustrate effects of treatment on (A) *Ctlc* mRNA expression, (B) *Gapdh* mRNA expression, (C) *Gusb* mRNA expression, (D) *Hprt* mRNA expression, (E) *Pgk1* mRNA expression, and (F) *Tubb5* mRNA expression. Data were analyzed using a two-way ANOVA followed by Fisher's LSD test using a single pooled error value, if appropriate, at a two-tailed alpha level of 0.05. *$p < 0.05$, **$p < 0.01$, ***$p < 0.001$, ****$p < 0.0001$. For all experimental conditions, there were 3 technical replicates ($n = 3$) from $N = 1$ experiment. Abbreviations: BBS, borate-buffered saline; CM, culture media; *Ctlc*, gene encoding clathrin heavy chain; *Gapdh*, gene encoding glyceraldehyde 3-phosphate dehydrogenase; *Gusb*, gene encoding beta glucuronidase; *Hprt*, gene encoding hypoxanthine-guanine phosphoribosyltransferase; LPS, lipopolysaccharide; *Pgk1*, gene encoding phosphoglycerate kinase; *Tubb*, gene encoding tubulin beta chain.

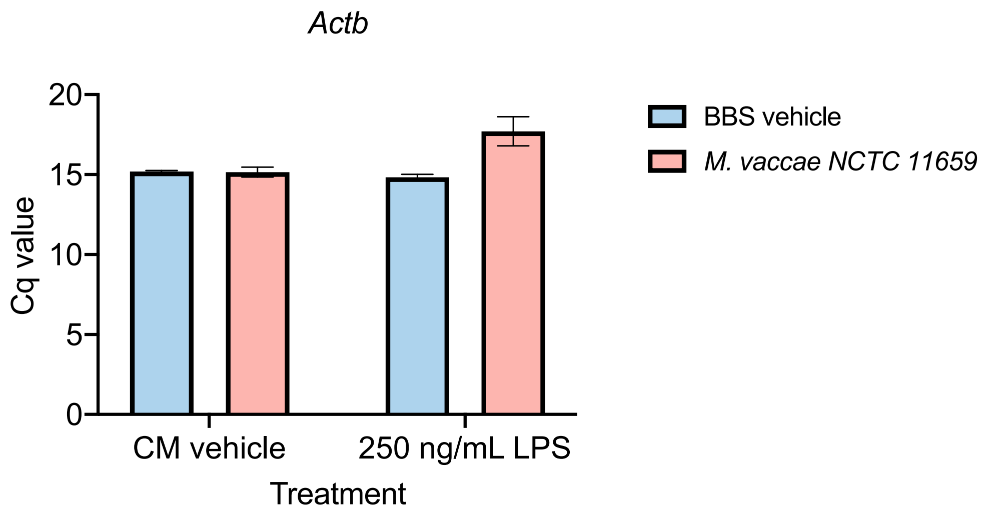

Figure S2. Effects of treatment of murine BV-2 microglial cells with *M. vaccae* NCTC 11659 (100 µg/mL or sterile borate-buffered saline vehicle control condition; 48 h) and lipopolysaccharide (0, 250 ng/mL; 24 h) or a cell culture media (CM) vehicle control condition on the expression of *Actb*. Abbreviations: BBS, borate-buffered saline. LPS, lipopolysaccharide.

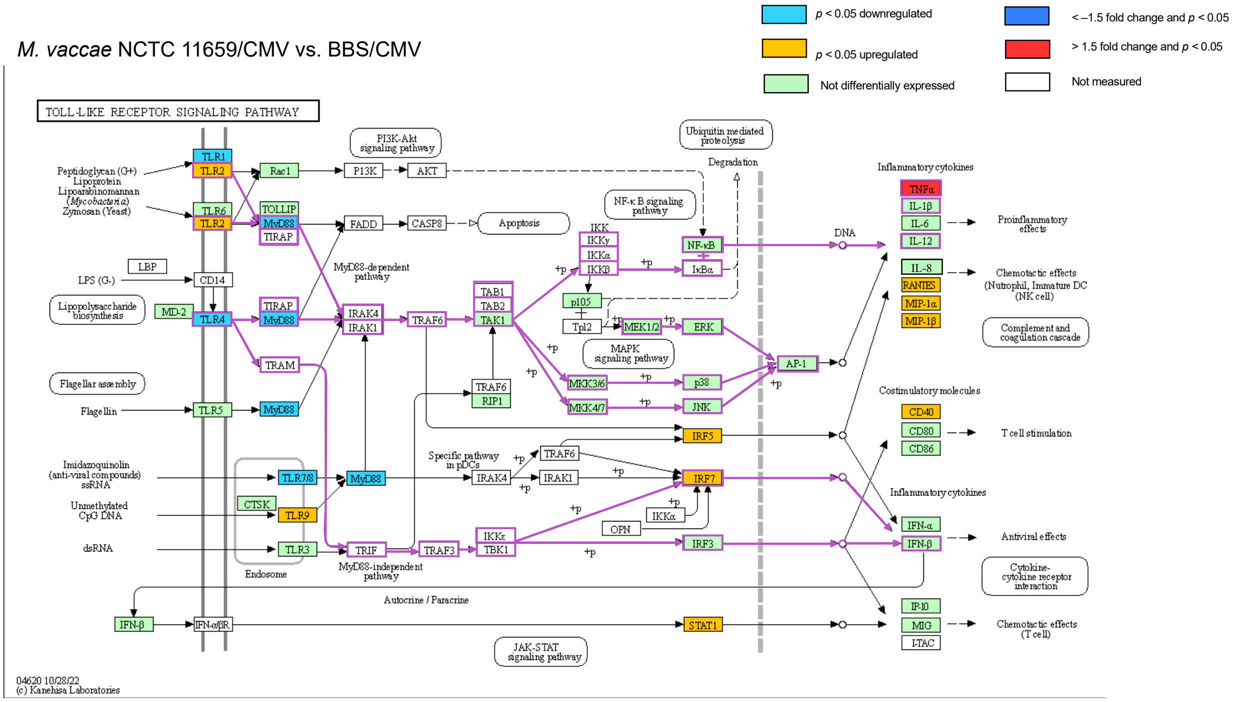

Figure S3. Toll-like receptor signaling KEGG pathway color-coded to reflect the effects of 100 µg/mL *M. vaccae* NCTC 11659 on gene expression. Genes highlighted in blue were downregulated by 100 µg/mL *M. vaccae* NCTC 11659, genes highlighted in red were upregulated by 100 µg/mL *M. vaccae* NCTC 11659, genes highlighted in green were not differentially expressed, and genes in white were not measured. Genes highlighted in light blue had a significant decrease in expression ($p < 0.05$) following treatment with 100 µg/mL *M. vaccae* NCTC 11659 but did not have a 1.5-fold change. Genes highlighted in orange had a significant increase in expression ($p < 0.05$) following treatment with 100 µg/mL *M. vaccae* NCTC 11659 but did not have a 1.5-fold change. The purple line indicates TLR2 and TLR4 signaling pathways.

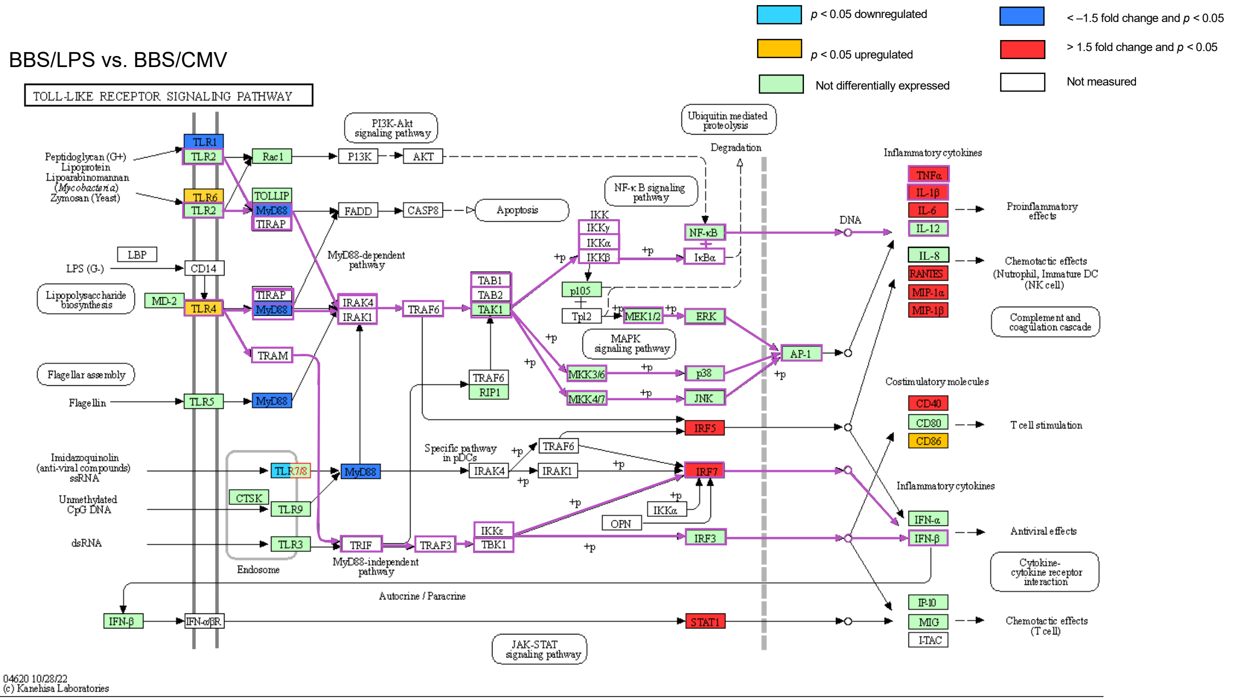

Figure S4. Toll-like receptor signaling KEGG pathway color-coded to reflect the effects of 250 ng/mL lipopolysaccharide (LPS) on gene expression. Genes highlighted in blue were downregulated by 250 ng/mL LPS and had at least a 1.5-fold change, genes highlighted in red were upregulated by 250 ng/mL LPS with at least a 1.5-fold change, genes highlighted in green were not differentially expressed, and genes in white were not measured. Genes highlighted in light blue had a significant decrease in expression ($p < 0.05$) following treatment with 250 ng/mL LPS treatment ($p < 0.05$) but did not have a 1.5-fold change. Genes highlighted in orange had a significant increase in expression ($p < 0.05$) following treatment with 250 ng/mL LPS treatment ($p < 0.05$) but did not have a 1.5-fold change. The purple line indicates TLR2 and TLR4 signaling pathways.

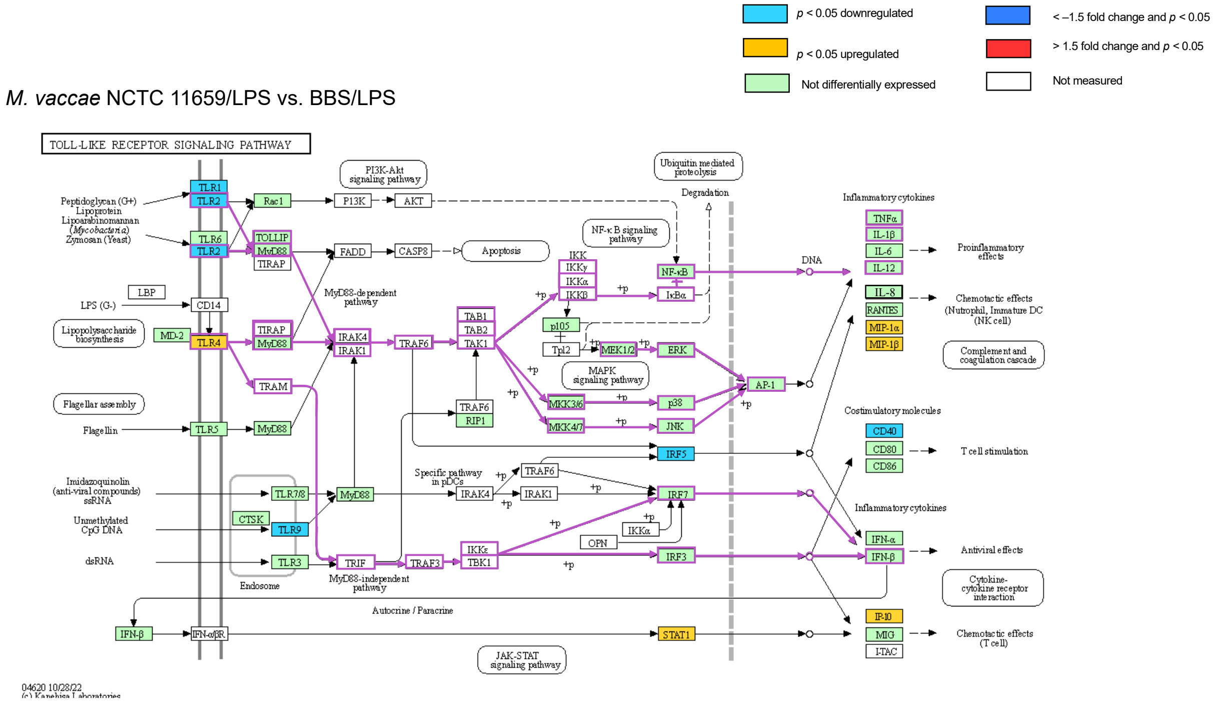

Figure S5. Toll-like receptor signaling KEGG pathway color-coded to reflect the effects of 100 µg/mL *M. vaccae* NCTC 11659 in the presence of 250 ng/mL lipopolysaccharide (LPS) on gene expression. Genes highlighted in blue were downregulated by 100 µg/mL *M. vaccae* NCTC 11659, genes highlighted in red were upregulated by 100 µg/mL *M. vaccae* NCTC 11659, genes highlighted in green were not differentially expressed, and genes in white were not measured. Genes highlighted in light-blue cells had a significant decrease in expression ($p < 0.05$) following treatment with 100 µg/mL *M. vaccae* NCTC 11659 ($p < 0.05$) but did not have a 1.5-fold change. Genes highlighted in orange had a significant increase in expression ($p < 0.05$) following treatment with 100 µg/mL *M. vaccae* NCTC 11659 ($p < 0.05$) but did not have a 1.5-fold change. The purple line indicates TLR2 and TLR4 signaling pathways.

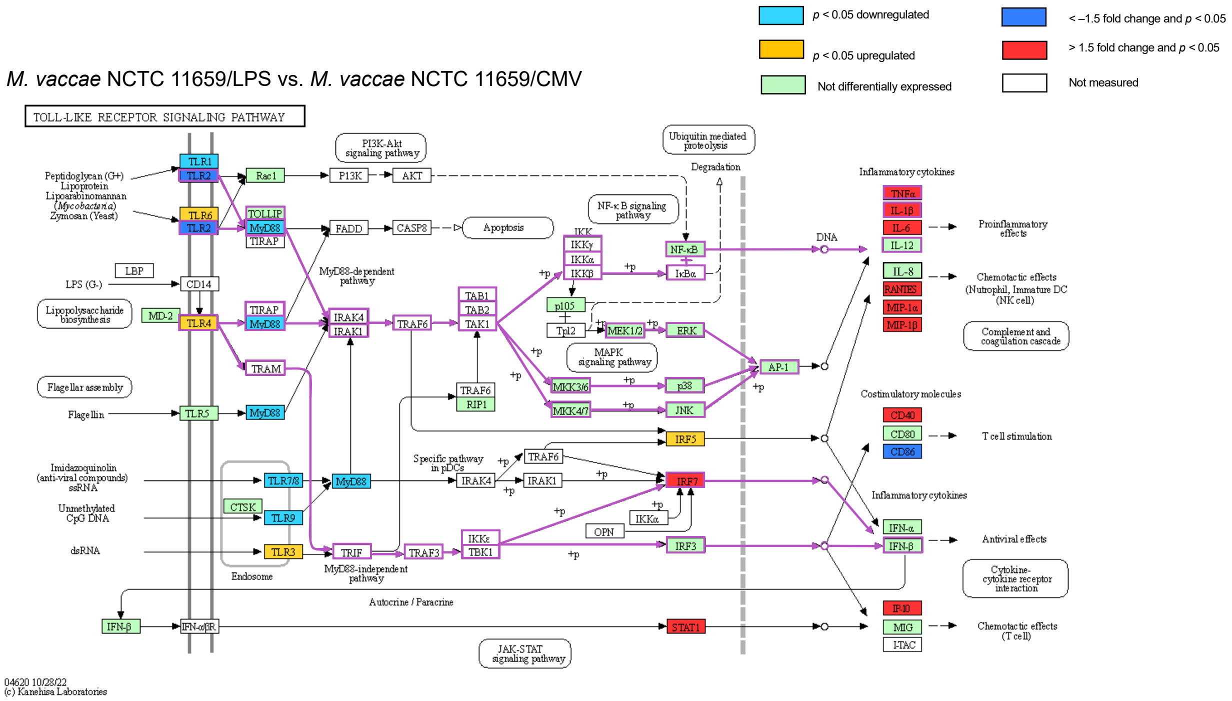

Figure S6. Toll-like receptor signaling KEGG pathway color-coded to reflect the effects of 250 ng/mL lipopolysaccharide (LPS) in the presence of 100 µg/mL *M. vaccae* NCTC 11659 on gene expression. Genes highlighted in blue were downregulated by 250 ng/mL LPS, genes highlighted in red were upregulated by 250 ng/mL LPS, genes highlighted in green were not differentially expressed, and genes in white were not measured. Genes highlighted in light blue had a significant decrease in expression ($p < 0.05$) following treatment with 250 ng/mL LPS treatment ($p < 0.05$) but did not have a 1.5-fold change. Genes highlighted in orange had a significant increase in expression ($p < 0.05$) following treatment with 250 ng/mL LPS treatment ($p < 0.05$) but did not have a 1.5-fold change. The purple line indicates TLR2 and TLR4 signaling pathways.

Supplemental tables

Table S1. Log2 fold changes among differentially expressed genes in the *M. vaccae* NCTC 11659/CMV versus BBS/CMV comparison.

Gene symbols	Log2 Fold Change
Upregulated	
C3	1.16881
Ccl2	0.698848
Tnf	0.677453
Downregulated	
Arg1	-1.64186
Smad7	-0.62623

Table S2. Genes that had *p*-values less than 0.05 in the *M. vaccae* NCTC 11659/CMV versus BBS/CMV comparison.

Gene Symbol	Log2 Fold Change
Upregulated	
C1ra	0.522863333
Map2k6	0.503656667
Relb	0.495151333
Tlr2	0.474013333
Il1a	0.45724
Tradd	0.441106667
Maff	0.42836
Ccl5	0.41674
Tnfaip3	0.413766667
Cd40	0.33927
Irf1	0.318105
Ccl3	0.304396667
Rock2	0.256366667
Ccl4	0.251118
Nfkb1	0.240120333
Rapgef2	0.234643333
Stat3	0.229596667
Mapkapk2	0.221023333
Irf7	0.20075
Il18	0.197
Cxcr1	0.194853333
Cfb	0.190016667
Nfe2l2	0.166356667

Tlr9	0.16627
Stat1	0.152266667
Ifit2	0.150683333
Itgb2	0.12703
Cebpb	0.118883333
Nod2	0.107374333
Grb2	0.10702
Cxcl2	0.096584
Shc1	0.09582
Mafg	0.093901333
Bcl2l1	0.092198
Mapk1	0.09019
Mapk8	0.086444
Hdac4	0.080853333
Mrc1	0.08044
Mx2	0.067417667
Mx1	0.066616667
Irf5	0.06568
Oasl1	0.05803
Gnaq	0.046157
Tollip	0.043597333
Ptgir	0.041338
Tgfb1	0.040803333
Hif1a	0.03787
Downregulated	
Il15	-0.48629
Oas2	-0.466773333
Fxyd2	-0.465263333
Ripk1	-0.407486667
Tlr1	-0.370292723
Cysltr1	-0.36564
Mef2d	-0.357553
Limk1	-0.339843333
Cxcr4	-0.321923333
Map3k5	-0.318477
Myd88	-0.292906667
Mafk	-0.292616667
Prkcb	-0.292127333
Mknk1	-0.272153333

Ptk2	-0.265663333
Il1rap	-0.255903333
Max	-0.243566667
Ddit3	-0.225653333
Hmgb1	-0.223086667
Ifi27l2a	-0.207843333
Stat2	-0.198543333
Ptgs1	-0.190846667
Trem2	-0.18747
Tlr8	-0.177774667
Traf2	-0.166396667
Cxcl10	-0.15852
Tlr4	-0.156593333
C3ar1	-0.15266
Cfl1	-0.150703333
Map2k4	-0.144073333
Hmgn1	-0.140913333
Tgfbr1	-0.135936667
Rela	-0.131246667
Jun	-0.12641
Rhoa	-0.11677
Nr3c1	-0.115952667
Tcf4	-0.115070333
Prkca	-0.114099
Pdgfa	-0.113133333
Fos	-0.109136667
Rac1	-0.10565
Tyrobp	-0.104916667
Keap1	-0.101661333
Gnas	-0.09738
Flt1	-0.094111
C4a	-0.086458333
Mef2c	-0.084173333
Gngt1	-0.071236667
Mapkapk5	-0.06905
Nfatc3	-0.068228
Ppp1r12b	-0.067863333
Raf1	-0.067411333
Map3k7	-0.065804

Gnb1	-0.063546667
Cdc42	-0.05227
Mmp9	-0.051743333
Nlrp3	-0.049161333
Ptgs2	-0.045203333
Pla2g4a	-0.021403993
Mapk3	-0.02038
Ly96	-0.018520667

Table S3. List of abbreviations for all gene symbols

Alox12, arachidonate 12-lipoxygenase
APC, antigen-presenting cell
Arg1, arginase 1
BBS, borate-buffered saline
Birc2, baculoviral IAP repeat-containing 2
C1qb, complement component 1, q subcomponent, beta polypeptide
C1ra, complement component 1, r subcomponent A
C1s1, complement component 1, s subcomponent 1
C3, complement component 3
Ccl2, C-C motif chemokine ligand 2
Ccl3, C-C motif chemokine ligand 3
Ccl4, C-C motif chemokine ligand 4
Ccl5, C-C motif chemokine ligand 5
Ccl7, C-C motif chemokine ligand 7
Ccr3, chemokine (C-C motif) receptor 3
Cd163, Cd163 antigen
Cd4, Cd4 antigen
Cd40, Cd40 antigen
Cebpb, CCAAT/enhancer binding protein, beta
Cfb, complement factor B
CMV, cell culture media vehicle
CNS, central nervous system
Csf1, colony-stimulating factor 1
Csf3, colony-stimulating factor 3
Cxcl10, C-X-C motif chemokine ligand 10
Cxcl2, C-X-C motif chemokine ligand 2
Cxcr1, chemokine (C-X-C motif) receptor 1
Cxcr4, chemokine (C-X-C motif) receptor 4
Daxx, fas death domain-associated protein
Ddit3, DNA-damage inducible transcript 3
Fasl, fas ligand; *Fos*, FBJ osteosarcoma oncogene
H2-eb1, histocompatibility 2, class II antigen E beta
Hif1a, hypoxia-inducible factor 1, alpha subunit
Hmgb1, high mobility group box 1
Hmgn1, high mobility group nucleosomal binding domain 1
Hspb1, heat shock protein 1
Ifi27l2A, interferon, alpha-inducible protein 27 like 2A
Ifi44, interferon-induced protein 44
Ifit1, interferon-induced protein with tetratricopeptide repeats 1
Ifit2, interferon-induced protein with tetratricopeptide repeats 2
Ifit3, interferon-induced protein with tetratricopeptide repeats 3
Iigp1, interferon-inducible GTPase 1
IL, interleukin
Il12b, interleukin 12b
Il18, interleukin 18

Il1a, interleukin 1 alpha
Il1b, interleukin 1 beta
Il1r1, interleukin 1 receptor, type 1
Il1rn, interleukin 1 receptor antagonist
Il23a, interleukin 23, alpha subunit p19
Il6, interleukin 6
Il6ra, interleukin 6 receptor, alpha
Irf1, interferon regulatory factor 1
Irf5, interferon regulatory factor 5
LPS, lipopolysaccharide
Lta, lymphotoxin A
Ly96, lymphocyte antigen 96
Maff, v-maf musculoaponeurotic fibrosarcoma oncogene family, protein F
M. vaccae, *Mycobacterium vaccae*
Mafg, v-maf musculoaponeurotic fibrosarcoma oncogene family, protein G
Map2k1, mitogen-activated protein kinase kinase 1
Mapkapk2, MAP kinase-activated protein kinase 2
Mef2a, myocyte enhancer factor 2a
Mef2c, myocyte enhancer factor 2C
Mmp9, matrix metallopeptidase 9
Mrc1, mannose receptor, C type 1
Mx1, MX dynamin-like GTPase 1
Mx2, MX dynamin-like GTPase 2
Myc, myelocytomatosis oncogene
Myd88, myeloid differentiation primary response gene 88
NCTC, National Collection of Type Cultures
Nfe2l2, nuclear factor, erythroid derived 2, like 2
Nfkb1, nuclear factor of kappa light polypeptide gene enhancer in B cells 1, p105
Nlrp3, NLR family, pyrin domain containing 3
Nod1, nucleotide-binding oligomerization domain containing 1
Nod2, nucleotide-binding oligomerization domain containing 2
Nos2, nitric oxide synthase 2, inducible
Nox1, NADPH oxidase 1
Oas1a, 2'-5' oligoadenylate synthetase 1a
Oas2, 2'-5' oligoadenylate synthetase 2
Oasl1, 2'-5' oligoadenylate synthetase-like 1
PNN, perineuronal net
Ptgir, prostaglandin I receptor (IP)
Ptgs1, prostaglandin-endoperoxide synthase 1
Ptgs2, prostaglandin-endoperoxide synthase 2
Rapgef2, rap guanine nucleotide exchange factor (GEF) 2
Rela, v-rel reticuloendotheliosis viral oncogene homolog A
Relb, avian reticuloendotheliosis viral (v-rel) oncogene related B
Ripk2, receptor (TNFRSF)-interacting serine-threonine kinase 2
Stat1, signal transducer and activator of transcription 1
Stat2, signal transducer and activator of transcription 2

Stat3, signal transducer and activator of transcription 3
TGFβ, transforming growth factor beta
TLR, toll-like receptor
Tlr1, toll-like receptor 1
Tlr2, toll-like receptor 2
Tlr3, toll-like receptor 3
Tlr6, toll-like receptor 6
Tlr8, toll-like receptor 8
Tlr9, toll-like receptor 9
Tnf, tumor necrosis factor
Tnfaip3, tumor necrosis factor, alpha-induced protein 3
Traf2, Tnf receptor-associated factor 2
Treg, regulatory T cell
Trem2, triggering receptor expressed on myeloid cells

Table S4. Log2 fold changes among differentially expressed genes in the BBS/250 ng/mL LPS versus BBS/CMV comparison.

Gene Symbol	Log2 Fold Change
Upregulated	
Cfb	5.60648
Ccl5	5.56549
Ifit1	4.024
Cd40	3.07338
Ifit3	2.99376
Il1b	2.79327
Il6	2.61309
Nos2	2.57817
Maff	2.55533
Tnf	2.4393
Tradd	2.29754
C3	2.26905
Ccl2	2.0218
Irf1	1.98211
Irf7	1.92943
Ccl3	1.85339
Rock2	1.57019
Cxcr1	1.56136
Oasl1	1.54032
Il1a	1.44444
Ccl4	1.37771
Map2k6	1.35468
Mx1	1.27039
Cxcl2	1.23382
Ifit2	1.13021
Tnfaip3	0.975547
C1ra	0.929432
Il18	0.830453
Il6ra	0.79481
Ifi44	0.756457
Csf1	0.719692
Irf5	0.713634
Stat1	0.70563
Mx2	0.702039
Keap1	0.671778

Ripk2	0.666244
Downregulated	
Arg1	-2.80374
Smad7	-1.93381
Limk1	-1.83436
Fxyd2	-1.79421
Ptgs1	-1.61283
Hmgn1	-1.53119
Trem2	-1.52992
Max	-1.5075
Myl2	-1.47985
Tlr8	-1.43853
Ripk1	-1.26378
Mef2d	-1.25684
Cysltr1	-1.25576
Hmgb1	-1.22569
C1qb	-1.15706
Cxcr4	-1.12752
Ptger4	-1.11958
Mafk	-0.999982
Ptk2	-0.996795
Mmp9	-0.985976
Mknk1	-0.943527
C1s1	-0.855773
Oas2	-0.85333
Cd86	-0.834836
Raf1	-0.834556
Map3k7	-0.819428
Pdgfa	-0.805386
Mrc1	-0.697027
Il1rap	-0.651947
Jun	-0.649737
Tlr1	-0.620468
Rhoa	-0.615622
Flt1	-0.610663
Cfl1	-0.602626
Myd88	-0.593948

Table S5. Genes that had *p*-values less than 0.05 in the BBS/LPS versus BBS/CMV comparison.

Gene Symbol	Log2 Fold Change
Upregulated	
Atf2	0.574514667
Cd4	0.548781333
Gnas	0.532883333
Grb2	0.530340667
Rac1	0.500567
Il10rb	0.494686667
Bcl6	0.492426667
H2-Eb1	0.476682667
Tcf4	0.427876667
Tyrobp	0.426743333
Mafg	0.41568
Mapk14	0.401537333
Map2k4	0.372703333
Map3k1	0.36534
Pla2g4a	0.360026333
Ppp1r12b	0.35498
Cdc42	0.338123333
Tlr7	0.331499333
Birc2	0.321431667
Gnaq	0.304983333
Gngt1	0.302697667
Mapkapk5	0.261233
Mapk1	0.236921
Gnb1	0.234823333
Tollip	0.224343333
C3ar1	0.211985333
Nr3c1	0.202471
Shc1	0.189009
Ly96	0.187679
Mapk3	0.158596667
Tlr4	0.145233333
Tgfb1	0.12621
Rela	0.100826667
Ifi27l2a	0.081143333
Nfe2l2	0.078463333

Stat2	0.04284
C1qa	0.024513333
Downregulated	
Ptgir	-0.528952333
Relb	-0.508803333
Elk1	-0.506823333
Map3k5	-0.48829
Nlrp3	-0.487453333
Ddit3	-0.473056667
Il1rn	-0.452563333
Map2k1	-0.436669667
Hdac4	-0.435948
Mapkapk2	-0.427086667
Cebpb	-0.422433667
Ccl7	-0.414796667
Nox1	-0.35121
Ptgs2	-0.3473188
Stat3	-0.31330722
Tlr9	-0.285496667
Hif1a	-0.224553333
Bcl2l1	-0.21676
Oas1a	-0.206146667
Nod1	-0.204346
Hmgb2	-0.18363
Nod2	-0.181143333
Mapk8	-0.16611
Rapgef2	-0.164476667
Myc	-0.147813
Tlr6	-0.132133333
Il1r1	-0.129887333
Nfatc3	-0.124976667
Prkca	-0.062955667
Fos	-0.024766667

Table S6. Log2 fold changes among differentially expressed genes in the *M. vaccae* NCTC 11659/250 ng/mL LPS versus BBS/250 ng/mL LPS condition.

Gene symbols	Log2 Fold Change
Upregulated	
Ccl7	1.10527
Csf3	0.711066
Downregulated	
Cysltr1	-0.630051

Table S7. Genes that had *p*-values less than 0.05 in the *M. vaccae* NCTC 11659/250 ng/mL LPS versus BBS/250 ng/mL LPS comparison.

Gene Symbol	Log2 Fold Change
Upregulated	
Mmp9	0.521552733
Tlr3	0.46441
Ccl2	0.454501667
Cxcr1	0.432406667
Rps6ka5	0.385053333
Fos	0.32347
Il15	0.32142
Rock2	0.321271
C1ra	0.295016667
Hdac4	0.293523333
Nos2	0.289856667
Ripk2	0.271563333
Ptgs2	0.257453333
Mafg	0.246756667
Mknk1	0.23039
Tradd	0.227473333
Il1rn	0.223083333
Myc	0.208986667
Cxcl2	0.191143333
Mapk8	0.191081
Fxyd2	0.185787467
Jun	0.185123333
Map2k4	0.184196667
Ccl4	0.168313333
Tcf4	0.167267667
Cxcl10	0.161426667
Flt1	0.140163333

Mrc1	0.1365
Ccl3	0.13141
Keap1	0.126113333
Stat1	0.11608
Mapk3	0.10595
Cfb	0.098346667
Oasl1	0.08909
Il1a	0.083816667
Pla2g4a	0.080459333
Shc1	0.076493333
Nlrp3	0.065152433
Tlr4	0.043196667
Downregulated	
Tlr9	-0.37297
Mx2	-0.2752792
Cd40	-0.252691667
Bcl6	-0.227363333
Tlr1	-0.225156667
Mx1	-0.219863333
Grb2	-0.206496667
Tnfaip3	-0.162046667
Mapk1	-0.155753333
Maff	-0.14415
Irf5	-0.13144
C3ar1	-0.130933333
Bcl2l1	-0.130643667
Atf2	-0.125656667
Tgfb1	-0.123026667
Il18	-0.12242
Tlr2	-0.122263333
Tyrobp	-0.113626667
C3	-0.110666667
Gnas	-0.089353333
Gnaq	-0.082727667
Il10rb	-0.075986667
Irf1	-0.075463333
Cfl1	-0.07277
Cdc42	-0.06998
Ly96	-0.05652

Oas1a	-0.053942667
Rela	-0.05245
Gnb1	-0.04362

Table S8. Log2 fold changes among differentially expressed genes in the *M. vaccae* NCTC 11659/250 ng/mL LPS versus *M. vaccae* NCTC 11659/CMV condition.

Gene symbol	Log2 Fold Change
Upregulated	
Cfb	5.51481
Ccl5	5.06203
Ifit1	4.22224
Ifit3	2.99882
Nos2	2.76852
Il6	2.70201
Il1b	2.67336
Cd40	2.48141
Tradd	2.08391
Maff	1.98282
Cxcr1	1.79891
Ccl2	1.77745
Irf7	1.76926
Tnf	1.72265
Ccl3	1.6804
Rock2	1.6351
Irf1	1.58854
Oasl1	1.57138
Ccl7	1.43047
Cxcl2	1.32839
Ccl4	1.29491
Il1a	1.07101
Ripk2	1.00964
Map3k5	1.00414
C3	0.989572
Mx1	0.983916
Keap1	0.899553
Il6ra	0.876298
Map2k6	0.873408
Ifit2	0.777177
Ifi44	0.757504
Il1rn	0.719116
C1ra	0.701586
Ddit3	0.697734

Il15	0.691392
Csf1	0.680683
Stat1	0.66944
Ptgs2	0.668
Hdac4	0.640543
Nlrp3	0.614881
Csf3	0.609804
Fos	0.591201
Downregulated	
Limk1	-1.59298
Cysltr1	-1.52017
Ptgs1	-1.4189
Myl2	-1.38455
Max	-1.33951
Trem2	-1.31826
Hmgn1	-1.31755
Smad7	-1.30721
C1qb	-1.25213
Arg1	-1.19992
Tlr8	-1.19457
Fxyd2	-1.14316
C1s1	-1.0975
Mef2d	-1.04409
Ptger4	-1.0306
Hmgb1	-0.968003
Ptk2	-0.947579
Ripk1	-0.848458
Map3k7	-0.846216
Cd86	-0.80308
Grb2	-0.801806
Raf1	-0.755615
Bcl6	-0.700543
Pdgfa	-0.685679
Cxcr4	-0.684825
Mafk	-0.642115
Mrc1	-0.640967
Atf2	-0.636656
H2-Eb1	-0.613079
Tlr2	-0.594341

Table S9. Genes that had *p*-values less than 0.05 in the *M. vaccae* NCTC 11659/250 ng/mL LPS versus *M. vaccae* NCTC 11659/CMV comparison.

Gene Symbol	Log2 Fold Change
Upregulated	
Elk1	0.553416667
Irf5	0.516513333
Map2k1	0.513035667
Il18	0.511036667
Prkcb	0.502143
Tlr3	0.502013333
Ptgir	0.4707
Rps6ka5	0.458906667
Myc	0.455173333
Prkca	0.453177
Tnfaip3	0.399733333
Cxcl10	0.37518
Mx2	0.359341467
Mapk8	0.341558
Nox1	0.323453333
Ifi27l2a	0.27288
Nfatc3	0.271963667
Oas1a	0.267537
Cebpb	0.267276667
Hif1a	0.263643333
Hmgb2	0.256328333
C4a	0.248158333
Stat2	0.228993333
Tlr4	0.224303333
Nod1	0.210726667
Mapkapk2	0.188243333
Traf2	0.16002
Rela	0.15726
Tlr6	0.122373667
C1qa	0.11133
Mapk3	0.101563333
Tgfbr1	0.088243333
Downregulated	

Mef2c	-0.564018667
Cd4	-0.538076667
Cfl1	-0.52469
Il10rb	-0.521076667
Gnas	-0.498796667
Rhoa	-0.491613333
Tlr1	-0.475331667
Rac1	-0.450123333
Mknk1	-0.440986667
Mapk14	-0.438436667
Tyrobp	-0.435796667
Myd88	-0.420828333
Mmp9	-0.412677
Mapk1	-0.412053333
Map3k1	-0.392781
Flt1	-0.376388333
Jun	-0.3382
Gnaq	-0.333230667
Oas2	-0.331556667
Tollip	-0.292194667
Mafg	-0.269578333
Cdc42	-0.242263333
Birc2	-0.21882
Pla2g4a	-0.211443893
Nfkb1	-0.196643
Tlr9	-0.18426
Tlr7	-0.154416667
Tcf4	-0.15361
Gnb1	-0.14455
Shc1	-0.144303333
Nfe2l2	-0.127403333
Tgfb1	-0.12099
Gngt1	-0.113633333
C3ar1	-0.110406667
Ly96	-0.100955
Mapkapk5	-0.10086

Table S10. Sample size for real-time reverse transcription polymerase chain reaction (RT-PCR).

**LPS Treatment**	**Bacterial Treatment**	**Sample Size (*n*)**
0 ng/mL	BBS	3
	100 µg/mL *M. vaccae* NCTC 11659	2
250 ng/mL	BBS	2
	100 µg/mL *M. vaccae* NCTC 11659	3

Table S11. Total RNA concentrations in each sample prior to assessment using the NanoString nCounter system.

**Sample Name**	**RNA concentration (ng/µl)**
Sample 1 (BBS1/CMV)	4.5
Sample 2 (BBS2/CMV)	2.5
Sample 3 (BBS3/CMV)	4.7
Sample 4 (Mv1/CMV)	4.4
Sample 5 (Mv2/CMV)	4.4
Sample 6 (Mv3/CMV)	4.1
Sample 7 (BBS/LPS1)	5.1
Sample 8 (BBS/LPS2)	5.1
Sample 9 (BBS/LPS3)	4.8
Sample 10 (Mv/LPS1)	4.9
Sample 11 (Mv/LPS2)	5.6
Sample 12 (Mv/LPS3)	4.9

Abbreviations: BBS, borate-buffered saline; CMV, culture media vehicle; LPS, lipopolysaccharide; Mv, *Mycobacterium vaccae* NCTC 11659.
